# Fast and parallel decomposition of constraint satisfaction problems

**DOI:** 10.1007/s10601-022-09332-1

**Published:** 2022-06-03

**Authors:** Georg Gottlob, Cem Okulmus, Reinhard Pichler

**Affiliations:** 1 University of Oxford, Oxford, UK; 2 TU Wien, Vienna, Austria

**Keywords:** Constraint satisfaction, Hypergraphs, Structural decomposition methods, Parallel computing

## Abstract

Constraint Satisfaction Problems (CSP) are notoriously hard. Consequently, powerful decomposition methods have been developed to overcome this complexity. However, this poses the challenge of actually computing such a decomposition for a given CSP instance, and previous algorithms have shown their limitations in doing so. In this paper, we present a number of key algorithmic improvements and parallelisation techniques to compute so-called Generalized Hypertree Decompositions (GHDs) faster. We thus advance the ability to compute optimal (i.e., minimal-width) GHDs for a significantly wider range of CSP instances on modern machines. This lays the foundation for more systems and applications in evaluating CSPs and related problems (such as Conjunctive Query answering) based on their structural properties.

## Introduction

Many real-life tasks can be effectively modelled as CSPs, giving them a vital importance in many areas of Computer Science. As solving CSPs is a classical NP-complete problem, there is a large body of research to find tractable fragments. One such line of research focuses on the underlying *hypergraph structure* of a CSP instance. A key result in this area is that CSP instances whose underlying hypergraph is acyclic, can be solved in polynomial time [41]. Several generalisations of acyclicity have been identified by defining various forms of hypergraph *decompositions*, each associated with a specific notion of *width* [8, 18]. Intuitively, the width measures how far away a hypergraph is from being acyclic, with a width of 1 describing the acyclic hypergraphs.

In this work, we focus on Generalized Hypertree Decompositions (GHD) [20], and generalized hypertree width (*ghw*). Formally, we look at the following problem: 
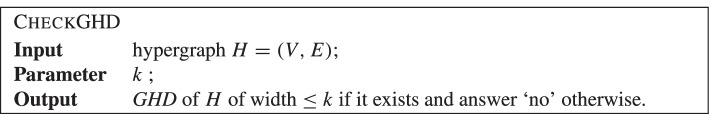


The computation of GHDs is itself intractable in the general case, already for width = 2 [15]. However, for (hypergraphs of) CSPs with realistic restrictions, this problem becomes tractable for a fixed parameter *k*. One such restriction is the bounded intersection property (BIP), which requires that any two constraints in a CSP only share a bounded number of variables [15]. Indeed, by examining a large number of CSPs from various benchmarks and real-life applications, it has been verified that this intersection of variables tends to be small in practice [14]. In that work, over 3,000 instances of hypergraphs of CSPs and also of Conjunctive Queries (CQs) were examined and made publicly available in the HyperBench benchmark at http://hyperbench.dbai.tuwien.ac.at.

The use of such decompositions can speed up the solving of CSPs and also the answering of CQs significantly. In fact, in [1] a speed-up up to a factor of 2,500 was reported for the CQs studied there. Structural decompositions are therefore already being used in commercial products and research prototypes, both in the CSP area as well as in database systems [1, 4, 5, 27, 33]. However, previous decomposition algorithms are limited in that they fail to find optimal decompositions (i.e., decompositions of minimal width) even for low widths. This is also the case for various GHD computation methods proposed in [14, 31, 38]. The overall aim of our work is therefore to advance the art of computing hypergraph decompositions and to make the use of GHDs for solving CSPs applicable to a significantly wider range of CSP instances than previous methods. More specifically, we derive the following research goals:

- Main Goal: Provide major improvements for computing hypergraph decompositions.

As part of this main goal, we define in particular:

- Sub-goal 1: Design novel parallel algorithms for structural decompositions, in particular GHDs, and
- Sub-goal 2: Put all this to work, by implementing and extensively evaluating these improvements.

Note that, apart from GHDs, there are also other types of hypergraph decompositions (see [18] for a comparison), notably tree decompositions (TDs) [37], hypertree decompositions (HDs) [19], and fractional hypertree decompositions (FHDs) [25]. TDs are the oldest and most intensively studied form of these decomposition methods, both in terms of their efficient computation (see e.g. [7, 29]), the potential for parallel algorithms (see e.g. [32]) and their application to a wide range of problems – including constraint solving [36]. However, compared with the other types of decompositions mentioned above, TDs have a serious drawback in the context of CSP evaluation and CQ answering: CSP and CQ algorithms based on any of these decompositions essentially run in time *O*(*n*^*k*^), where *n* is the size of the problem instance and *k* is the width of the decomposition used. However, if we have relations of arity *α*, then the treewidth may be up to a factor *α* worse than the width notions based on the other decomposition methods. This is why HDs, GHDs, and FHDs are the better choice for CSPs and CQs.

There exist systems for computing HDs [14, 23, 38], GHDs [14, 31, 38], and FHDs [13]. However, to the best of our knowledge, none of them makes use of parallelism. In theory, HD-computation is easiest among the three. Indeed, the check problem (i.e., the problem of checking if a decomposition of some fixed width exists and, in the positive case, computing such a decomposition) is tractable for HDs [19] but intractable for GHDs and FHDs even for width = 2 [15, 20]. Nevertheless, despite this tractability result, HD-computation has turned out to be computationally expensive in practice. And parallelisation of the computation is tricky in this case since, in contrast to GHDs and FHDs, HDs are based on a *rooted* tree. This makes it impossible to reuse the ideas of the parallel GHD computation applied here, which recursively splits the task of computing the tree underlying a GHD into subtrees which then have to be re-rooted appropriately when they are stitched together. FHDs are computationally yet more expensive than GHDs. So GHDs are a good middle ground among these 3 types of decompositions.

As a first step in pursuing the first goal, we aim at *generally applicable* simplifications of hypergraphs to speed up the decomposition of hypergraphs. Here, “general applicability” means that these simplifications can be incorporated into any decomposition algorithms such as the ones presented in [13, 14] and also earlier work such as [23]. Moreover, we aim at heuristics for guiding the decomposition algorithms to explore more promising parts of the big search space first.

However, it will turn out that these simplifications and heuristics are not sufficient to overcome a principal shortcoming of existing decomposition algorithms, namely their sequential nature. Modern computing devices consist of multi-core architectures, and we can observe that single-core performance has mostly stagnated since the mid-2000s. So to produce programs which run optimally on modern machines, one must find a way of designing them to run efficiently in parallel. However, utilising multi-core systems is a non-trivial task, which poses several challenges. In our design of parallel GHD-algorithms, we focus on three key issues:

i minimising synchronisation delay as much as possible,
ii finding a way to partition the search space equally among CPUs, and thus utilising the resources optimally,
iii supporting efficient backtracking, a key element of all structural decomposition algorithms presented so far.

In order to evaluate our algorithmic improvements and our new parallel GHD-algorithms, we have implemented them and tested them on the publicly available HyperBench benchmark mentioned above. For our implementation, we decided to use the programming language Go proposed by Google [10], which is based on the classical Communication Sequential Processes pattern by [28], since it reduces the need for explicit synchronisation.

To summarise, the **main results** of this work are as follows:

- We have developed three parallel algorithms for computing GHDs, where the first two are loosely based on the *balanced separator* method from [3, 14]. As has been mentioned above, none of the previous systems for computing HDs, GHDs, or FHDs makes use of parallelism. Our parallel approach has opened the way for a *hybrid approach*, which combines the strengths of parallel and sequential algorithms. This hybrid approach ultimately proved to be the best.
- In addition to designing parallel algorithms, we propose several algorithmic improvements such as applying multiple pre-processing steps on the input hypergraphs and using various heuristics to guide the search for a decomposition. While most of the pre-processing steps have already been used before, their combination and, in particular, a proof that their exhaustive application yields a unique normal form (up to isomorphism) is new. Moreover, for the hybrid approach, we have explored when to best switch from one approach to the other.
- We have implemented the parallel algorithms together with all algorithmic improvements and heuristics presented here. The source code of the program is available under https://github.com/cem-okulmus/BalancedGo. With our new algorithms and their implementation, dramatically more instances from HyperBench could be solved compared with previous algorithms. More specifically, we could extend the number of hypergraphs with exact *ghw* known by over 50%. In total, this means that for over 75% of all instances of HyperBench, the exact *ghw* is now known. If we leave aside the randomly-generated CSPs, and focus on the those from real world applications, we can show an increase of close to 100%, thus almost doubling the number of instances solved.

Our work therefore makes it possible to compute GHDs efficiently on modern machines for a wide range of CSPs. It enables the fast recognition of low widths for many instances encountered in practice (as represented by HyperBench) and thus lays the foundation for more systems and applications in evaluating CSPs and CQs based on their structural properties.

The remainder of this paper is structured as follows: In Section 2, we provide the needed terminology and recall previous approaches. In Section 3, we present our general algorithmic improvements. This is followed by a description of our parallelisation strategy in Section 4. Experimental evaluations are presented in Section 5. In Section 6, we summarise our main results and highlight directions for future work. This paper is an enhanced and extended version of work presented at IJCAI-PRICAI 2020 [21].

## Preliminaries

### CSPs & hypergraphs

A *constraint satisfaction problem* (CSP) *P* is a set of *constraints*(*S*_*i*_,*R*_*i*_) with 1 ≤ *i* ≤ *m*, where each $S_{i} = \{ s_{0}, {\dots } s_{n} \}$ is a set of variables and *R*_*i*_ a constraint relation which contains tuples of size *n* using values from a domain *D*. A solution to *P* is a mapping of variables to values from the domain *D*, such that for each constraint we map the variables to some tuple in its constraint relation.

A *hypergraph* *H* is a tuple (*V* (*H*),*E*(*H*)), consisting of a set of vertices *V* (*H*) and a set of hyperedges (synonymously, simply referred to as “edges”) $E(H) \subseteq 2^{V(H)}$,

where the notation 2^*V* (*H*)^ signifies the power set over *V* (*H*). To get the hypergraph of a CSP *P*, we consider *V* (*H*) to be the set of all variables in *P*, to be precise $\bigcup _{i} S_{i}$, and each *S*_*i*_ to be one hyperedge. Here, we disregard the constraint relations, as they contain no additional structural information.

Recall that solving a CSP corresponds to model checking a first-order formula *Φ* (representing the constraints *S*_*i*_) over a finite structure (made up by the relations *R*_*i*_) such that the only connectives allowed in *Φ* are ∃ and ∧, whereas ∀,∨, and ¬ are disallowed. Hence, formally, CSP solving is equivalent to answering conjunctive queries (CQs) in the database world [30, 35]. In the sequel, we will mainly concentrate on CSPs with the understanding that all our results equally apply to CQs.

The *intersection size* of a hypergraph *H* is defined as the minimum integer *c*, such that for any two edges *e*_1_,*e*_2_ ∈ *E*(*H*), *e*_1_ ∩ *e*_2_ ≤ *c*. A class ${{\mathscr{C}}}$ of hypergraphs has the *bounded intersection property (BIP)*, if there exists a constant *c* such that every hypergraph $H \in {{\mathscr{C}}}$ has intersection size ≤ *c*.

We are frequently dealing with sets of sets of vertices (e.g., sets of edges). For $S \subseteq 2^{V(H)}$, we write $\bigcup S$ and $\bigcap S$ as a short-hand for taking the union or intersection, respectively, of this set of sets of vertices, i.e., for $S = \{s_{1}, \dots , s_{\ell }\}$, we have $\bigcup S = {\bigcup }_{i=1}^{\ell } s_{i}$ and $\bigcap S = \bigcap _{i=1}^{\ell } s_{i}$. For a set *S* of edges, we will alternatively also write *V* (*S*) to denote the vertices contained in any of the edges in *S*. That is, we have $V(S) = \bigcup S$.

### Decompositions

A *generalized hypertree decomposition (GHD)* of a hypergraph *H* = (*V* (*H*),*E*(*H*)) is a tuple 〈*T*,*χ*,*λ*〉, where *T* = (*N*,*E*(*T*)) is a tree, and *χ* and *λ* are labelling functions, which map to each node *n* ∈ *N* two sets, $\chi (n) \subseteq V(H)$ and $\lambda (n) \subseteq E(H)$. For a node *n* we call *χ*(*n*) the *bag*, and *λ*(*n*) the *edge cover* of *n*. We denote with *B*(*λ*(*n*)) the set {*v* ∈ *V* (*H*)∣*v* ∈ *e* for some *e* ∈ *λ*(*n*)}, i.e., the set of vertices “covered” by *λ*(*n*). The functions *χ* and *λ* have to satisfy the following conditions:

1. For each *e* ∈ *E*(*H*), there is a node *n* ∈ *N* s.t. $e \subseteq \chi (n)$.
2. For each vertex *v* ∈ *V* (*H*), {*n* ∈ *N*∣*v* ∈ *χ*(*n*)} is a connected subtree of *T*.
3. For each node *n* ∈ *N*, we have that $\chi (n) \subseteq B(\lambda (n))$.

The second condition is also referred to as the *connectedness condition*. The *width of a GHD* is defined as $\max \limits \{|\lambda (n)| \mid n \in N \}$. The generalized hypertree width (ghw) of a hypergraph is the smallest width of any of its GHDs. Deciding if *ghw*(*H*) ≤ *k* for a hypergraph *H* and fixed *k* is NP-complete, as one needs to consider exponentially many possible choices for the bag *χ*(*n*) for a given edge cover *λ*(*n*).

It was shown in [14] that for any class of hypergraphs enjoying the BIP, one only needs to consider a polynomial set of subsets of hyperedges (called *subedges* ) to compute their *ghw*. This fact will be explained in more detail in Section 2.2.

### Example 1

**Fig. 1:**
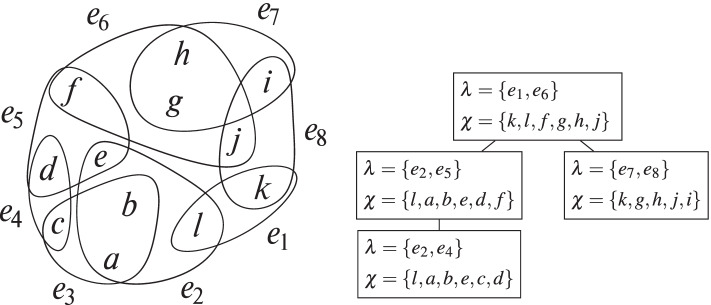
An example hypergraph, where the vertices are represented by letters, with explicit edge names, together with a GHD of width 2

An example of a hypergraph is shown in Fig. 1, as well as a GHD of this hypergraph. We can see that no *λ*-label uses more than two hyperedges, and thus this GHD has width 2, and the *ghw* of the hypergraph is also ≤ 2. In fact, the hypergraph contains alpha cycles [12], e.g., {*e*_2_,*e*_3_,*e*_4_,*e*_5_}. Hence, we also know its *ghw* must be > 1. Taken together, its *ghw* is therefore exactly 2.

### Components & separators

Consider a set of vertices $W \subseteq V(H)$. A set of edges $C \subseteq E(H)$ is [*W*]*-connected* if for any two distinct edges $e,e^{\prime } \in C$ there exists a sequence of vertices $v_{1},\dots ,v_{h}$ and a sequence of edges $e_{0}, \dots , e_{h}$ (*h* ≥ 1) with *e*_0_ = *e* and $e_{h} = e^{\prime }$ such that *v*_*i*_ ∈ *e*_*i*− 1_ ∩ *e*_*i*_ and *v*_*i*_∉*W* for each *i* ∈{1,…,*h*}. In other words, there is a path from *e* to $e^{\prime }$ which only goes through vertices outside *W*. A set $C \subseteq E(H)$ is a [*W*]*-component*, if *C* is maximal [*W*]-connected. For a set of edges $S \subseteq E(H)$, we say that *C* is “[*S*]*-connected*” or an “[*S*]*-component*” as a short-cut for *C* is “[*W*]-connected” or a “[*W*]-component”, respectively, with $W = \bigcup S$. We also call *S* a *separator* in this context. The *size of an* [*S*]*-componentC* is simply its cardinality. For a hypergraph *H* and a set of edges $S \subseteq E(H)$, we say that *S* is a *balanced separator* if all [*S*]-components of *H* have size $\leq \frac {|E(H)|}{2}$.

### Example 2

**Fig. 2:**
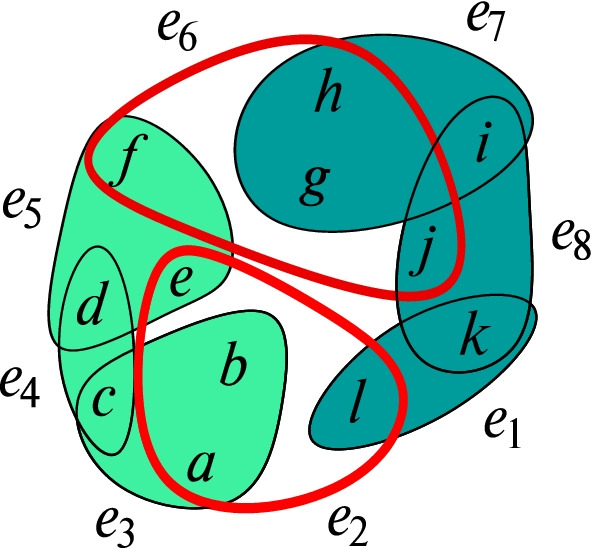
Connected components and their respective separator, visually marked

An example for a separator that generates multiple connected components can be seen in Fig. 2. The separator *S* consists of two hyperedges *e*_2_,*e*_6_, marked by thicker edges. The corresponding [*S*]-components *C*_1_ = {*e*_3_,*e*_4_,*e*_5_} and *C*_2_ = {*e*_1_,*e*_7_,*e*_8_} are highlighted visually.

It was shown in [2] that, for every GHD 〈*T*,*χ*,*λ*〉 of a hypergraph *H*, there exists a node *n* ∈ *N* such that *λ*(*n*) is a balanced separator of *H*. This property can be used when searching for a GHD of size *k* of *H*, as we shall recall in Section 2.2 below.

### Computing hypertree decompositions (HDs)

We briefly recall the basic principles of the det-*k*-decomp program from [23] for computing Hypertree Decompositions (HDs), which was the first implementation of the original HD algorithm from [19]. HDs are GHDs with an additional condition to make their computation tractable in a way explained next.

For fixed *k* ≥ 1, det-*k*-decomp tries to construct an HD of a hypergraph *H* in a top-down manner. It thus maintains a set *C* of edges, which is initialised to *C* := *E*(*H*). For a node *n* in the HD (initially, this is the root of the HD), it “guesses” an edge cover *λ*(*n*), i.e., $\lambda (n) \subseteq E(H)$ and |*λ*(*n*)|≤ *k*. For fixed *k*, there are only polynomially many possible values *λ*(*n*). det-*k*-decomp then proceeds by determining all [*λ*(*n*)]-components *C*_*i*_ with $C_{i} \subseteq C$. The additional condition imposed on HDs (compared with GHDs) restricts the possible choices for *χ*(*n*) and thus guarantees that the [*λ*(*n*)]-components inside *C* and the [*χ*(*n*)]-components inside *C* coincide. This is the crucial property for ensuring polynomial time complexity of HD-computation – at the price of possibly missing GHDs with a lower width.

Now let $C_{1}, \dots , C_{\ell }$ denote the [*λ*(*n*)]-components with $C_{i} \subseteq C$. By the maximality of components, these sets $C_{i} \subseteq E(H)$ are pairwise disjoint. Moreover, it was shown in [19] that if *H* has an HD of width ≤ *k*, then it also has an HD of width ≤ *k* such that the edges in each *C*_*i*_ are “covered” in different subtrees below *n*. More precisely, this means that *n* has *ℓ* child nodes $n_{1}, \dots , n_{\ell }$, such that for every *i* and every *e* ∈ *C*_*i*_, there exists a node *n*_*e*_ in the subtree rooted at *n*_*i*_ with $e \subseteq \chi (n_{e})$. Hence, det-*k*-decomp recursively searches for an HD of the hypergraphs *H*_*i*_ with *E*(*H*_*i*_) = *C*_*i*_ and $V(H_{i}) = \bigcup C_{i}$ with the slight extra feature that also edges from *E*(*H*) ∖ *C*_*i*_ are allowed to be used in the *λ*-labels of these HDs.

### Computing GHDs

It was shown in [15] that, even for fixed *k* = 2, deciding if *ghw*(*H*) ≤ *k* holds for a hypergraph *H* is NP-complete. However, it was also shown there that if a class of hypergraphs satisfies the BIP, then the problem becomes tractable. The main reason for the NP-completeness in the general case is that, for a given edge cover *λ*(*n*), there can be exponentially many bags *χ*(*n*) satisfying condition 3 of GHDs, i.e., $\chi (n)\subseteq B(\lambda (n))$. In other words, to have a sound and complete procedure to check if a given hypergraph has *ghw* at most *k*, one would need to check exponentially many possible bags for any given edge cover.

Now let $\lambda (n) = \{e_{i_{1}}, \dots , e_{i_{\ell }}\}$ with *ℓ* ≤ *k*. Of course, if we restrict each $e_{i_{j}}$ to the subedge $e^{\prime }_{i_{j}} = e_{i_{j}} \cap \chi (n)$ and define $\lambda ^{\prime }(n) = \{e^{\prime }_{i_{1}}, \dots , e^{\prime }_{i_{\ell }}\}$, then we get $\chi (n) = B(\lambda ^{\prime }(n))$. The key to the tractability shown in [15] in case of the BIP (i.e., the intersection of any two distinct edges is bounded by a constant *b*) is twofold: first, it is easy to see that w.l.o.g., we may restrict the search for a GHD of desired width *k* to so-called “bag-maximal” GHDs. That is, for any node *n*, it is impossible to add another vertex to *χ*(*n*) without violating a condition from the definition of GHDs. And second, it is then shown in [15] for bag-maximal GHDs, that each $e^{\prime }_{i_{j}}$ is either equal to $e_{i_{j}}$ or a subset of $e_{i_{j}}$ with $|e^{\prime }_{i_{j}}| \leq k\cdot b$. Hence, there are only polynomially many choices of subedges $e^{\prime }_{i_{j}}$ and also of *χ*(*n*). More precisely, for a given edge *e*, the set of subedges to consider is defined as follows:

1 $$f_{e}(H,k) = \bigcup\limits_{e_{1},\dots,e_{j} \in (E(H) \setminus \{e\}), j \leq k} 2^{(e \cap (e_{1} \cup {\dots} \cup e_{j}))} )$$

In [14], this property was used to design a program for GHD computation as a straightforward extension of det-*k*-decomp by adding the *polynomially many* subedges *f*_*e*_(*H*,*k*) for all *e* ∈ *E*(*H*) to *E*(*H*). In the hypergraph extended in this way, we can thus be sure that *λ*(*n*) can always be replaced by $\lambda ^{\prime }(n)$ with $\chi (n) = B(\lambda ^{\prime }(n))$.

In [14], yet another GHD algorithm was presented. It is based on the use of *balanced separators* and extends ideas from [3]. The motivation of this approach comes from the observation that there is no useful upper bound on the size of the subproblems that have to be solved by the recursive calls of the det-*k*-decomp algorithm. In fact, for some node *n* with corresponding component *C*, let $C_{1}, \dots , C_{\ell }$ denote the [*λ*(*n*)]-components with $C_{i} \subseteq C$. Then there may exist an *i* such that *C*_*i*_ is “almost” as big as *C*. In other words, in the worst case, the recursion depth of det-*k*-decomp may be linear in the number of edges.

The Balanced Separator approach from [14] uses the fact that every GHD must contain a node whose *λ*-label is a balanced separator. Hence, in each recursive decomposition step for some subset $E^{\prime }$ of the edges of *H*, the algorithm “guesses” a node $n^{\prime }$ such that $\lambda (n^{\prime })$ is a balanced separator of the hypergraph with edges $E^{\prime }$. Of course, this node $n^{\prime }$ is not necessarily a child node *n*_*i*_ of the current node *n* but may lie somewhere inside the subtree *T*_*i*_ below *n*. However, since GHDs can be arbitrarily rooted, one may first compute this subtree *T*_*i*_ with $n^{\prime }$ as the root and with *n*_*i*_ as a leaf node. This subtree is then (when returning from the recursion) connected to node *n* by rerooting *T*_*i*_ at *n*_*i*_ and turning *n*_*i*_ into a child node of *n*. The definition of balanced separators guarantees that the recursion depth is logarithmically bounded. This makes the Balanced Separator algorithm a good starting point for our parallel algorithm to be presented in Section 4.

## Algorithmic improvements

In this section, we present several algorithmic improvements of decomposition algorithms. We start with some simplifications of hypergraphs, which can be applied as a preprocessing step for any hypergraph decomposition algorithm, i.e., they are not restricted to the GHD algorithms discussed here. We shall then also mention further algorithmic improvements which are specific to the GHD algorithms presented in this paper. We note that, while the GHD-specific algorithmic improvements are new, the simplifications mentioned below have already been used before and/or are quite straightforward. We prove that their exhaustive application to an arbitrary hypergraph yields a unique normal form up to isomorphism. For the sake of completeness, we also prove the correctness and polynomial time complexity of their application.

### Hypergraph preprocessing

An important step to speed up decomposition algorithms is the simplification of the input hypergraph. Before we formally present such a simplification, we observe that we may restrict ourselves to *connected* hypergraphs, formally those having only a single [*∅*]-component, since a GHD of a hypergraph consisting of several connected components can be obtained by combining the GHDs of each connected component in an “arbitrary” way, e.g., appending the root of one GHD as a child of an arbitrarily chosen node of another GHD. This can never violate the connectedness condition, since the GHDs of different components have no vertices in common. It is easy to verify that the simplifications proposed below never make a connected hypergraph disconnected. Hence, splitting a hypergraph into its connected components can be done upfront, once and for all. After that, we are exclusively concerned with connected hypergraphs. Given a (connected) hypergraph *H* = (*V* (*H*),*E*(*H*)), we thus propose the exhaustive application of the following reduction rules in a don’t-care non-deterministic fashion:

The so-called GYO reduction was introduced in [24, 42] to test the acyclicity of a hypergraph. It consists of the Rules 1 and 2 recalled below:

**Rule 1.**: Suppose that *H* contains a vertex *v* that only occurs in a single edge *e*. Then we may delete *v* from *e* and thus from *V* (*H*) altogether.
**Rule 2.**: Suppose that *H* contains two edges *e*_1_,*e*_2_, such that $e_{1} \subseteq e_{2}$. Then we may delete *e*_1_ from *E*(*H*).
  The next reduction of hypergraphs makes use of the notion of *types* of vertices. Here the *type* of a vertex *v* is defined as the set of edges *e* which contain *v*. We thus define Rule 3 as follows:
**Rule 3.**: Suppose that *H* contains vertices *v*_1_,*v*_2_ of the same *type*. Then we may delete *v*_2_ from *V* (*H*) and thus from all edges containing *v*_2_.
  The next reduction rule considered here uses the notion of *hinges*. In [26], hinge decompositions were introduced to help split CSPs into smaller subproblems. In [17], the combination of hinge decompositions and hypertree decompositions was studied. We also make use of hinge decompositions as part of our preprocessing. More specifically, we define the following reduction rule:
**Rule 4.**: Let *e* ∈ *E*(*H*) and let ${{\mathscr{C}}} = \{C_{1}, \dots , C_{\ell }\}$ with *ℓ* ≥ 2 denote the [*e*]-components of *H*. Then we may split *H* into hypergraphs *H*_1_ = (*V* (*H*_1_),*E*(*H*_1_)),…, *H*_*ℓ*_ = (*V* (*H*_*ℓ*_),*E*(*H*_*ℓ*_)) with *H*(*E*_*i*_) = *C*_*i*_ ∪{*e*} and $V(H_{i}) = \bigcup E(H_{i})$ for each *i*.
  The above simplifications (above all the splitting into smaller hypergraphs via Rule 4) may produce a hypergraph that is so small that the construction of a GHD of width ≤ *k* for given *k* ≥ 1 becomes trivial. The following rule allows us to eliminate such trivial cases:
**Rule 5.**: If $\lvert {E(H)}\rvert \leq k$, then *H* may be deleted. It has a trivial GHD consisting of a single node *n* with *λ*(*n*) = *E*(*H*) and $\chi (n) = \bigcup E(H)$.

In Theorems 1 and 2 below, we state several crucial properties of the reductions. Most importantly, these reductions neither add nor lose solutions. Moreover, preprocessing a hypergraph with these rules can be done in polynomial time.

Note that, even though all Rules 1 – 5 are applied to a *single hypergraph*, the result in case of Rule 4 is a *set of hypergraphs*. Hence, strictly speaking, these rules form a rewrite system that transforms a set of hypergraphs into another set of hypergraphs, where the starting point is a singleton consisting of the initial hypergraph only. However, to keep the notation simple, we will concentrate on the effect of these rules on a single hypergraph with the understanding that application of one of these rules comes down to selecting an element from a set of hypergraphs and replacing this element by the hypergraph(s) according to the above definition of the rules.

#### Theorem 1

Preprocessing an input hypergraph *H* via Rules 1 – 5 is sound. More precisely, let $\{H_{1}, \dots , H_{m}\}$ be the result of exhaustive application of Rules 1 – 5 to a hypergraph *H*. Then, for any *k* ≥ 1, we have *ghw*(*H*) ≤ *k* if and only if, for every $i \in \{1, \dots , m\}$, *ghw*(*H*_*i*_) ≤ *k* holds.

As for the complexity, this transformation of *H* into $\{H_{1}, \dots , H_{\ell }\}$ is feasible in polynomial time. Moreover, any collection of GHDs of width ≤ *k* of $H_{1}, \dots , H_{\ell }$ can be transformed in polynomial time into a GHD of *H* of width ≤ *k*.

#### Proof

We split the proof in two main parts: first, we consider the complexity of exhaustive application of Rules 1 – 5 and then we prove the soundness of the rules. The polynomial-time complexity of constructing a GHD of *H* from GHDs of the resulting hypergraphs $\{H_{1}, \dots , H_{m}\}$ will be part of the correctness proof. □

#### Complexity of exhaustive rule application

Rules 1 – 3 have the effect that the size of *H* is strictly decreased by either deleting vertices or edges. Hence, there can be only linearly many applications of Rules 1 – 3 and each of these rule applications is clearly feasible in polynomial time. Likewise, Rule 5, which allows us to delete a non-empty hypergraph, can only be applied linearly often and any application of this rule is clearly feasible in polynomial time. Checking if Rule 4 is applicable and actually applying Rule 4 is also feasible in polynomial time. Hence, it only remains to show that the total number of applications of Rule 4 is polynomially bounded. To see this, we first of all make the following observation on the number of edges in each *H*_*i*_: consider a single application of Rule 4 and suppose that, for some edge *e*, there are *ℓ* [*e*]-components $C_{1},\dots , C_{\ell }$. These [*e*]-components are pairwise disjoint and we have $C_{i} \subseteq E(H) \setminus \{e\}$ for each *i*. Hence, if $\lvert {E(H)}\rvert = n$ and $\lvert {C_{i}}\rvert = n_{i}$ with *n*_*i*_ ≥ 1, then $n_{1} + {\dots } + n_{\ell } \leq n -1$ holds. Moreover, $\lvert {E(H_{i})}\rvert = n_{i} +1$, since we add *e* to each component. We claim that, in total, when applying Rules 1 – 4 exhaustively to a hypergraph *H* with *n* ≥ 3 edges, there can be at most 2*n* − 3 applications of Rule 4. Note that for *n* = 1 or *n* = 2, Rule 4 is not applicable at all.

We prove this claim by induction on *n*: if *H* has 3 edges, then an application of Rule 4 is only possible, if we find an edge *e*, such that there are 2 [*e*]-components *C*_1_,*C*_2_, each consisting of a single edge. Hence, such an application of Rule 4 produces two hypergraphs *H*_1_,*H*_2_ with 2 edges each, to which no further application of Rule 4 is possible. Hence, the total number of applications of Rule 4 is bounded by 1 and, for *n* = 3, we indeed have 1 ≤ 6 − 3 ≤ 2*n* − 3.

For the induction step, suppose that the claim holds for any hypergraph with ≤ *n* − 1 edges and suppose that *H* has *n* edges. Moreover, suppose that an application of Rule 4 for some edge *e* is possible with *ℓ* ≥ 2 [*e*]-components $C_{1}, \dots , C_{\ell }$ and let $\lvert {C_{i}}\rvert = n_{i}$. Then *H* is split into *ℓ* hypergraphs $H_{1}, \dots , H_{\ell }$ with $\lvert {E(H_{i})}\rvert = n_{i} +1$. Note that applications of any of the Rules 1 – 3 to the hypergraphs *H*_*i*_ can never increase the number of edges. These rules may thus be ignored and we may apply the induction hypothesis to each *H*_*i*_. Hence, for every *i*, there are at most 2(*n*_*i*_ + 1) − 3 = 2*n*_*i*_ − 1 applications of Rule 4 in total possible for *H*_*i*_. Taking all the resulting hypergraphs $H_{1}, \dots , H_{\ell }$ together, the total number of applications of Rule 4 is therefore $\leq (2 n_{1} + {\dots } + 2 n_{\ell }) - \ell$. Together with the inequalities $n_{1} + {\dots } + n_{\ell } \leq n-1$ and *ℓ* ≥ 2, and adding the initial application of Rule 4, we thus have, in total, ≤ 2(*n* − 1) − *ℓ* + 1 = 2*n* − 2 − *ℓ* + 1 ≤ 2*n* − 2 − 2 + 1 = 2*n* − 3 applications of Rule 4.

#### Soundness

For the soundness of our reduction system, we have to prove the soundness of each single rule application. Likewise, for the polynomial-time complexity of constructing a GHD of *H* from the GHDs of the final hypergraph set $\{H_{1}, \dots , H_{m}\}$, it suffices to show that one can efficiently construct a GHD of the original hypergraph from the GHD(s) of the hypergraph(s) resulting from a single rule application. This is due to the fact that we have already shown above that the total number of rule applications is polynomially bounded. It thus suffices to prove the following claim:

#### Claim A

Let *H* be a hypergraph and suppose that $H^{\prime }$ is the result of a single application of one of the Rules 1 – 3 to *H*. Then *ghw*(*H*) ≤ *k* if and only if ${ghw}(H^{\prime }) \leq k$. Moreover, in the positive case, a GHD of *H* of width ≤ *k* can be constructed from a GHD of $H^{\prime }$ of width ≤ *k* in polynomial time.

Likewise, suppose that $H_{1}, \dots , H_{\ell }$ is the result of a single application of Rule 4 to *H*. Then *ghw*(*H*) ≤ *k* if and only if, for every $i \in \{1, \dots , \ell \}$, *ghw*(*H*_*i*_) ≤ *k* holds. Moreover, in the positive case, a GHD of *H* of width ≤ *k* can be constructed from GHDs of $H_{1}, \dots , H_{\ell }$ of width ≤ *k* in polynomial time.

Note that we have omitted Rule 5 in this claim, since both the soundness and the polynomial-time construction of a GHD of width ≤ *k* are trivial. The proof of Claim A is straightforward but lengthy due to the case distinction over the 4 remaining rules. It is therefore deferred to Section 3.3.

Note that the application of one rule may enable the application of another rule; so their combination may lead to a greater simplification compared to just any one rule alone. Now the question naturally arises if the order in which we apply the rules has an impact on the final result. We next show that exhaustive application of Rules 1 – 5 leads to a unique (up to isomorphism) result, even if they are applied in a don’t-care non-deterministic fashion.

#### Theorem 2

Transforming a given hypergraph with Rules 1 – 5 leads to a *unique normal form*. That is, let *H* be a hypergraph and let $\{H_{1}, \dots , H_{m}\}$ be the result of exhaustively applying Rules 1 – 5. Then $\{H_{1}, \dots , H_{m}\}$ is unique (up to isomorphism) no matter in which order the Rules 1 – 5 are applied.

#### Proof

Recall that, in Theorem 1, we have already shown that the rewrite system is terminating (actually, we have even shown that there are at most polynomially many rule applications). In order to show that the rewrite system guarantees a unique normal form (up to isomorphism), it is therefore sufficient to show that it is *locally confluent* [6]. That is, we have to prove the following property: Let ${{\mathscr{H}}}$ be a set of hypergraphs and suppose that there are two possible ways of applying Rules 1 – 5 to (an element *H* of) ${{\mathscr{H}}}$, so that ${{\mathscr{H}}}$ can be transformed to either ${{\mathscr{H}}}_{1}$ or ${{\mathscr{H}}}_{2}$. Then there exists a set of hypergraphs ${{\mathscr{H}}}^{\prime }$, such that both ${{\mathscr{H}}}_{1}$ and ${{\mathscr{H}}}_{2}$ can be transformed into ${{\mathscr{H}}}^{\prime }$ by a sequence of applications of Rules 1 – 5. In the notation of [6], this property is succinctly presented as follows:

$${\mathscr{H}}_{1} \leftarrow {\mathscr{H}} \rightarrow {\mathscr{H}}_{2} \ \ \Rightarrow \ \ {\mathscr{H}}_{1} \downarrow {\mathscr{H}}_{2}$$

To prove this property, we have to consider all possible pairs (*i*,*j*) of applicable Rules *i* and *j*.

This case disctinction is rather tedious (especially the cases where Rule 4 is involved) but not difficult. We thus defer the details to Section 3.4.

### Finding balanced separators fast

It has already been observed in [23] that the ordering in which edges are considered is vital for finding an appropriate edge cover *λ*(*n*) for the current node *n* in the decomposition fast. However, the ordering used in [23] for det-*k*-decomp, (which was called MCSO, i.e., maximal cardinality search ordering) turned out to be a poor fit for finding balanced separators. A natural alternative was to consider, for each edge *e*, all possible paths between vertices in the hypergraph *H*, and how much the length of these paths increases after removal of *e*. This provides a weight for each edge, based on which we can define the *maximal separator ordering*. In our tests, this proved to be a very effective heuristic. Unfortunately, computing the maximal separator ordering requires solving the all-pairs shortest path problem. Using the well-known Floyd-Warshall algorithm [16, 40] as a subroutine, this leads to a fairly high complexity – see Table 1 – which proved to be prohibitively expensive for practical instances. We thus explored two other, computationally simpler, heuristics, which order the edges in descending order of the following measures:

- The *vertex degree* of an edge *e* is defined as ${\sum }_{v \in e} deg (v)$, where *deg*(*v*) denotes the degree of a vertex *v*, i.e., the number of edges containing *v*.
- The *edge degree* of an edge *e* is |{*f* : *e* ∩ *f*≠*∅*}|, i.e., the number of edges *e* has a non-empty intersection with.

In our empirical evaluation, we found both of these to be useful compromises between speeding up the search for balanced separators and the complexity of computing the ordering itself, with the vertex degree ordering yielding the best results, i.e., compute *λ*(*n*) by first trying to select edges with higher vertex degree.

**Table 1:** Overview of the complexity of the four methods considered for ordering hyperedges

Method of edge ordering	Runtime worst case complexity
Maximal cardinality search ordering	*O*(|*E*(*H*)|^2^)
Maximal separator ordering	*O*(|*E*(*H*)|⋅|*V* (*H*)|^3^)
Vertex degree ordering	*O*(|*E*(*H*)|^2^)
Edge degree ordering	*O*(|*E*(*H*)|^2^)

#### Finding the next balanced separator

Finding a balanced separator fast is important for the performance of our GHD algorithm, but it is not enough: if the balanced separator thus found does not lead to a successful GHD computation, we have to try another one. Hence, it is important to find the next balanced separator fast and to avoid trying the same balanced separator multiple times. The GHD algorithm based on balanced separators presented in [14] searches through all *ℓ*-tuples of edges (with *ℓ* ≤ *k*) to find the next balanced separator. The number of edge-combinations thus checked is $${\sum }_{i=1}^{k}\left (\begin {array}{cc} {N}\\{i} \end {array}\right )$$, where *N* denotes the number of edges. Note that this number of edges is actually higher than in the input hypergraph due to the subedges that have to be added for the tractability of GHD computation (see Section 2.2). Before we explain our improvement, let us formally explain how subedges factor into the search. Let us assume that we are given an edge cover $(e_{1}, \dots , e_{k})$, consisting of exactly *k* edges. Using the function *f*_*e*_(*H*,*k*) which generates the set of subedges to consider for any given edge *e*, defined in Section 2.2, we get the following set of edge combinations when factoring in all the relevant subedges:

$$\{(e^{\prime}_{1}, \dots, e^{\prime}_{k}) \mid e^{\prime}_{i} \in \{ e_{i} \cup f_{e_{i}}(H,k) \}, 1 \leq i \leq k \}$$

We note a significant source of redundancy in this set. If one only focuses on the combination of *l* ≤ *k* edges to intersect with *e*, it is possible that the same bags (when taking the union of their vertices) can be generated multiple times

We can address this by shifting our focus on the actual bags *χ*(*n*) generated from each *λ*(*n*) thus computed. Therefore, we initially only look for balanced separators of size *k*, checking $$\left (\begin {array}{cc} {N}\\{k} \end {array}\right )$$ many initial choices of *λ*(*n*). Only if a choice of *λ*(*n*) and $\chi (n) = \bigcup \lambda (n)$ does not lead to a successful recursive call of the decomposition procedure, we also inspect subsets of *χ*(*n*) – strictly avoiding the computation of the same subset of *χ*(*n*) several times by inspecting different subedges of the original edge cover *λ*(*n*). We thus also do not add subedges to the hypergraph upfront but only as they are needed as part of the backtracking when the original edge cover *λ*(*n*) did not succeed. Separators consisting of fewer edges are implicitly considered by allowing also the empty set as a possible subedge.

#### Summary

Our initial focus was to speed up existing decomposition algorithms via improvements as described above. However, even though these algorithmic improvements showed some positive effect, it turned out that a more fundamental change is needed. We have thus turned our attention to parallelisation, which will be the topic of Section 4. But first we present the missing parts of the proofs of Theorems 1 and 2 in Sections 3.3 and 3.4, respectively.

### Completion of the proof of theorem 1

It remains to prove Claim A from the proof in Section 3.1.

#### Proof Proof of the Claim

We prove the claim for each rule separately. It is convenient to treat *E*(*H*) as a multiset, i.e., *E*(*H*) may contain several “copies” of an edge. This simplifies the argumentation below, when the deletion of vertices may possibly make two edges identical. Note that, if at all, this only happens in intermediate steps, since Rule 2 above will later lead to the deletion of such copies anyway. □

#### Rule 1

*H* = (*V* (*H*),*E*(*H*)) contains a vertex *v* that only occurs in a single edge *e* and we delete *v* from *e* and from *V* (*H*) altogether. Let $e^{\prime } = e \setminus \{v\}$. Then $H^{\prime } = (V(H^{\prime }), E(H^{\prime }))$ with $V(H^{\prime }) = V(H) \setminus \{v\}$ and $E(H^{\prime }) = (E(H) \setminus \{e\}) \cup \{e^{\prime }\}$.⇒: Let ${\mathscr{D}} = \langle T, \chi , \lambda \rangle$ be a GHD of *H* of width ≤ *k*. We construct GHD ${\mathscr{D}}^{\prime } = \langle T^{\prime }, \chi ^{\prime }, \lambda ^{\prime } \rangle$ as follows: the tree structure *T* remains unchanged, i.e., we set = *T*. For every node *n* in the tree $T^{\prime }$, we define $\lambda ^{\prime }(n)$ and $\chi ^{\prime }(n)$ as follows:

- If *e* ∈ *λ*(*n*), then $$\lambda ^{\prime }(n) = (\lambda (n) \setminus \{e\}) \cup \{e^{\prime }\}$$.
- If *v* ∈ *χ*(*n*), then $$\chi ^{\prime }(n) = \chi (n) \setminus \{v\}$$.
- For all other nodes *n* in $T^{\prime }$, we set $\lambda ^{\prime }(n) = \lambda (n)$ and $\chi ^{\prime }(n) = \chi (n)$.

It is easy to verify that ${\mathscr{D}}^{\prime }$ is a GHD of $H^{\prime }$. Moreover, the width clearly does not increase by this transformation.⇐: Let ${\mathscr{D}}^{\prime } = \langle T^{\prime }, \chi ^{\prime }, \lambda ^{\prime } \rangle$ be a GHD of $H^{\prime }$ of width ≤ *k*. By the definition of GHDs, $T^{\prime }$ must contain at least one node *n*, such that $e^{\prime } \subseteq \chi ^{\prime }(n)$. We arbitrarily choose one such node $\hat {n}$ with $e^{\prime } \subseteq \chi ^{\prime }(\hat {n})$. Then we construct GHD ${\mathscr{D}} = \langle T, \chi , \lambda \rangle$ as follows:

- *T* contains all nodes and edges from $T^{\prime }$ plus one additional leaf node $n^{\prime }$ which we append as a child node of $\hat {n}$.
- For $n^{\prime }$, we set $\lambda (n^{\prime }) = \{ e\}$ and $\chi (n^{\prime }) = e$.
- Let *n* be a node in $T^{\prime }$ with $e^{\prime } \in \lambda ^{\prime }(n)$. Then we set $\lambda (n) = (\lambda ^{\prime }(n) \setminus \{e^{\prime }\}) \cup \{e\}$ and we leave $\chi ^{\prime }$ unchanged, i.e., $\chi (n) = \chi ^{\prime }(n)$.
- For all other nodes *n* in *T*, we set $\lambda (n) = \lambda ^{\prime }(n)$ and $\chi (n) = \chi ^{\prime }(n)$.

Clearly, ${\mathscr{D}}$ can be constructed from ${\mathscr{D}}^{\prime }$ in polynomial time. Moreover, it is easy to verify that ${\mathscr{D}}$ is a GHD of *H*. In particular, the connectedness condition is not violated by the introduction of the new node $n^{\prime }$ into the tree, since vertex $v \in \chi (n^{\prime })$ occurs nowhere else in ${\mathscr{D}}$ and all other vertices in $\chi (n^{\prime })$ are also contained in $\chi (\hat {n})$ for the parent node $\hat {n}$ of $n^{\prime }$. Moreover, the width clearly does not increase by this transformation since the new node $n^{\prime }$ has $\lvert {\lambda (n^{\prime })}\rvert = 1$ and for all other *λ*-labels, the cardinality has been left unchanged.

#### Rule 2

Suppose that *H* = (*V* (*H*),*E*(*H*)) contains two edges *e*_1_,*e*_2_, such that $e_{1} \subseteq e_{2}$ and we delete *e*_1_ from *E*(*H*), i.e., $H^{\prime } = (V(H^{\prime }), E(H^{\prime }))$ with $V(H^{\prime }) = V(H)$ and $E(H^{\prime }) = E(H) \setminus \{e_{1}\})$.⇒: Let ${\mathscr{D}} = \langle T, \chi , \lambda \rangle$ be a GHD of *H* of width ≤ *k*. We construct GHD ${\mathscr{D}}^{\prime } = \langle T^{\prime }, \chi ^{\prime }, \lambda ^{\prime } \rangle$ as follows: the tree structure *T* remains unchanged, i.e., we set $T^{\prime } = T$. For every node *n* in the tree $T^{\prime }$, we define $\lambda ^{\prime }(n)$ and $\chi ^{\prime }(n)$ as follows:

- If *e*_1_ ∈ *λ*(*n*), then $$\lambda ^{\prime }(n) = (\lambda (n) \setminus \{e_{1}\}) \cup \{e_{2}\}$$.
- For all other nodes *n* in $T^{\prime }$, we set $\lambda ^{\prime }(n) = \lambda (n)$.
- For all nodes *n* in $T^{\prime }$, we set $\chi ^{\prime }(n) = \chi (n)$.

It is easy to verify that ${\mathscr{D}}^{\prime }$ is a GHD of $H^{\prime }$ and that the width does not increase by this transformation.⇐: Let ${\mathscr{D}}^{\prime } = \langle T^{\prime }, \chi ^{\prime }, \lambda ^{\prime } \rangle$ be a GHD of $H^{\prime }$ of width ≤ *k*. It is easy to verify that then ${\mathscr{D}}^{\prime }$ is also a GHD of *H*. Indeed, we only need to verify that $T^{\prime }$ contains a node *n* with $e_{1} \subseteq \chi ^{\prime }(n)$. By the definition of GHDs, there exists a node *n* in $T^{\prime }$ with $e_{2} \subseteq \chi ^{\prime }(n)$. Hence, since we have $e_{1} \subseteq e_{2}$, also $e_{1} \subseteq \chi ^{\prime }(n)$ holds.

#### Rule 3

Suppose that *H* = (*V* (*H*),*E*(*H*)) contains two vertices *v*_1_,*v*_2_ which occur in precisely the same edges and we delete *v*_2_ from all edges and thus from *V* (*H*) altogether, i.e., $H^{\prime } = (V(H^{\prime }), E(H^{\prime }))$ with $V(H^{\prime }) = V(H) \setminus \{v_{2}\}$ and $E(H^{\prime }) = \{e \setminus \{v_{2}\} \mid e \in E(H) \}$.

It is convenient to introduce the following notation: suppose that $E(H) = \{ e_{1}, \dots , e_{\ell }\}$. Then we denote $E(H^{\prime })$ as $E(H^{\prime }) = \{ e^{\prime }_{1}, \dots , e^{\prime }_{\ell }\}$, where $e^{\prime }_{i} = e_{i} \setminus \{v_{2}\}$. Of course, we have $e^{\prime }_{i} = e_{i}$ whenever *v*_2_∉*e*_*i*_.⇒: Let ${\mathscr{D}} = \langle T, \chi , \lambda \rangle$ be a GHD of *H* of width ≤ *k*. We construct GHD ${\mathscr{D}}^{\prime } = \langle T^{\prime }, \chi ^{\prime }, \lambda ^{\prime } \rangle$ as follows: the tree structure *T* remains unchanged, i.e., we set $T^{\prime } = T$. For every node *n* in the tree $T^{\prime }$, we define $\lambda ^{\prime }(n)$ and $\chi ^{\prime }(n)$ as follows:

- Suppose that $\lambda (n) = \{ e_{i_{1}}, \dots , e_{i_{j}}\}$ for some *j* ≤ *k*. Then we set $\lambda ^{\prime }(n) = \{ e^{\prime }_{i_{1}}, \dots , e^{\prime }_{i_{j}}\}$.
- For all nodes *n* in $T^{\prime }$, we set $\chi ^{\prime }(n) = \chi (n) \setminus \{v_{2}\}$.

It is easy to verify that ${\mathscr{D}}^{\prime }$ is a GHD of $H^{\prime }$ and that the width does not increase by this transformation.⇐: Let ${\mathscr{D}}^{\prime } = \langle T^{\prime }, \chi ^{\prime }, \lambda ^{\prime } \rangle$ be a GHD of $H^{\prime }$ of width ≤ *k*. Then we construct GHD ${\mathscr{D}} = \langle T, \chi , \lambda \rangle$ as follows: the tree structure $T^{\prime }$ remains unchanged, i.e., we set $T = T^{\prime }$. For every node *n* in the tree *T*, we define *λ*(*n*) and *χ*(*n*) as follows:

- Suppose that $\lambda ^{\prime }(n) = \{ e^{\prime }_{i_{1}}, \dots , e^{\prime }_{i_{j}}\}$ for some *j* ≤ *k*. Then we set $\lambda (n) = \{ e_{i_{1}}, \dots , e_{i_{j}}\}$.
- For all nodes *n* in $T^{\prime }$ with $v_{1} \in \chi ^{\prime }(n)$, we set $\chi (n) = \chi ^{\prime }(n) \cup \{v_{2}\}$.
- For all other nodes *n* in $T^{\prime }$, we set *χ*(*n*) = *χ*(*n*)^′^.

Clearly this transformation is feasible in polynomial time and it does not increase the width. In order to show that ${\mathscr{D}}$ is indeed a GHD of *H*, there are two non-trivial parts, namely: (1) for every *e*_*α*_ ∈ *E*(*H*), there exists a node *n* in *T* with $e_{\alpha } \subseteq \chi (n)$ and (2) $\chi (n) \subseteq B(\lambda (n))$ holds for every node *n* even if we add vertex *v*_2_ to the *χ*-label. These are the two places where we make use of the fact that *v*_1_ and *v*_2_ occur in precisely the same edges in *E*(*H*).

For part (1), note that there exists a node *n* in $T^{\prime }$ (and hence in *T*), such that $e^{\prime }_{\alpha } \subseteq \chi ^{\prime }(n)$. If $v_{1} \not \in \chi ^{\prime }(n)$, then $v_{1} \not \in e^{\prime }_{\alpha }$ and, therefore *v*_1_∉*e*_*α*_. Hence, (since *v*_1_ and *v*_2_ have the same type) also *v*_2_∉*e*_*α*_. We thus have $e_{\alpha } = e^{\prime }_{\alpha }$ and $e_{\alpha } \subseteq \chi (n) = \chi ^{\prime }(n)$. On the other hand, if $v_{1} \in \chi ^{\prime }(n)$, then *v*_2_ ∈ *χ*(*n*) by the above construction of ${\mathscr{D}}$. Hence, $e_{\alpha } \subseteq \chi (n)$ again holds, since $e_{\alpha } \subseteq e^{\prime }_{\alpha } \cup \{v_{2}\}$.

For part (2), consider an arbitrary vertex *v* ∈ *χ*(*n*). We have to show that *v* ∈ *B*(*λ*(*n*)). First, suppose that *v*≠*v*_2_. Then we have $v \in \chi ^{\prime }(n) \subseteq B(\lambda ^{\prime }(n)) \subseteq B(\lambda (n))$. It remains to consider the case *v* = *v*_2_. Then, by the above construction of ${\mathscr{D}}$, we have $v_{1} \in \chi ^{\prime }(n)$. We observe the following chain of implications: $v_{1} \in \chi ^{\prime }(n)$ ⇒ $v_{1} \in e^{\prime }_{\alpha }$ for some $e^{\prime }_{\alpha }\in \lambda ^{\prime }(n)$ ⇒ *v*_1_ ∈ *e*_*α*_ for some *e*_*α*_ ∈ *λ*(*n*) ⇒ (since *v*_1_ and *v*_2_ have the same type) *v*_2_ ∈ *e*_*α*_ for some *e*_*α*_ ∈ *λ*(*n*). That is, *v* ∈ *B*(*λ*(*n*)) indeed holds.

#### Rule 4

Suppose that *H* = (*V* (*H*),*E*(*H*)) contains an edge *e* with [*e*]-components $C_{1}, \dots , C_{\ell }$ with *ℓ* ≥ 2. Further, suppose that we apply Rule 4 to replace *H* by the hypergraphs $H_{1} = (V(H_{1}), E(H_{1})),\dots , H_{\ell } = (V(H_{\ell }), E(H_{\ell }))$ with *H*(*E*_*j*_) = *C*_*j*_ ∪{*e*} and $V(H_{j}) = \bigcup E(H_{j})$ for each *j*.

⇒: Let ${\mathscr{D}} = \langle T, \chi , \lambda \rangle$ be a GHD of *H* of width ≤ *k*. We construct GHDs ${\mathscr{D}}_{j} = \langle T_{j}, \chi _{j}, \lambda _{j} \rangle$ of each *H*_*j*_ as follows: by the definition of GHDs, there must be a node *n* in *T* such that $e \subseteq \chi (n)$ holds. We choose such a node *n* and, w.l.o.g., we may assume that *n* is the root of ${{\mathscr{D}}}$. Let $\{D_{1}, \dots , D_{m}\}$ denote the [*χ*(*n*)]-components of *H*. It was shown in [19], that ${\mathscr{D}}$ can be transformed into a GHD ${\mathscr{D}}^{\prime } = \langle T^{\prime }, \chi ^{\prime }, \lambda ^{\prime } \rangle$, such that the root node *n* is left unchanged (i.e., in particular, we have $\chi (n) = \chi ^{\prime }(n)$ and $\lambda (n) = \lambda ^{\prime }(n)$) and *n* has *m* child nodes $n_{1}, \dots , n_{m}$, such that there is a one-to-one correspondence between these child nodes and the $[\chi ^{\prime }(n)]$-components $D_{1}, \dots , D_{m}$ in the following sense: for every edge *e*_*i*_ ∈ *D*_*i*_, there exists a node $n^{\prime }_{i}$ in the subtree rooted at *n*_*i*_ in $T^{\prime }$ such that $e_{i} \subseteq \chi ^{\prime }(n^{\prime }_{i})$. Intuitively, this means that the subtrees rooted at each of the child nodes of *n* “cover” precisely one $[\chi ^{\prime }(n)]$-component. We make the following crucial observations:

1. For every $[\chi ^{\prime }(n)]$-component *D*_*i*_, there exists a unique [*e*]-component *C*_*j*_, such that $D_{i} \subseteq C_{j}$. This is due to the fact that every $[\chi ^{\prime }(n)]$-connected set of edges is also [*e*]-connected, since $e \subseteq \chi ^{\prime }(n)$.
2. Let $D_{0} = \{f \in E(H) \mid f \subseteq \chi ^{\prime }(n)\}$. Then *E*(*H*) is partitioned into *D*_0_,*D*_1_, $\dots , D_{m}$. That is $D_{0} \cup D_{1} \cup {\dots } \cup D_{m} = E(H)$ and *D*_*i*_ ∩ *D*_*j*_ = *∅* for every pair *i*≠*j* of indices. This property can be seen as follows: every edge *f* ∈ *E*(*H*) with $f \not \subseteq \chi ^{\prime }(n)$ must be contained in some $[\chi ^{\prime }(n)]$-component. Hence, $D_{0} \cup D_{1} \cup {\dots } \cup D_{m} = E(H)$ clearly holds. On the other hand, by the very definition of components, any two distinct $[\chi ^{\prime }(n)]$-components *D*_*i*_,*D*_*j*_ with *i*≠*j* and *i*,*j* ≥ 1, are disjoint. Finally, also *D*_0_ and any *D*_*i*_ with *i* ≥ 1 are disjoint since an edge *f* with $f \subseteq \chi ^{\prime }(n)$ cannot be $[\chi ^{\prime }(n)]$-connected with any other edge.

Then, for $j \in \{1, \dots , \ell \}$, we define a GHD ${\mathscr{D}}_{j} = \langle T_{j}, \chi _{j}, \lambda _{j} \rangle$ of *H*_*j*_ as follows:

- *T*_*j*_ is the subtree of $T^{\prime }$ consisting of the following nodes:
  - the root node *n* is contained in *T*_*j*_;
  - for every $i \in \{1, \dots , m\}$, if $D_{i} \subseteq C_{j}$, then all nodes in the subtree rooted at *n*_*i*_ are contained in *T*_*j*_;
  - no further nodes are contained in *T*_*j*_.
- For every node $\hat {n}$ in *T*_*j*_, we set $\chi _{j}(\hat {n}) = \chi ^{\prime }(\hat {n}) \cap V(H_{j})$.
- For every node $\hat {n}$ in *T*_*j*_, we distinguish two cases for defining $\lambda _{j}(\hat {n})$:
  - If $\lambda ^{\prime }(\hat {n}) \subseteq E(H_{j})$ holds, then we set $\lambda _{j}(\hat {n}) = \lambda ^{\prime }(\hat {n})$.
  - If $\lambda ^{\prime }(\hat {n}) \not \subseteq E(H_{j})$ holds, then $\delta = \lambda ^{\prime }(\hat {n}) \setminus E(H_{j}) \neq \emptyset$ holds. In this case, we set $\lambda _{j}(\hat {n}) = (\lambda ^{\prime }(\hat {n}) \setminus \delta ) \cup \{e \}$.

It remains to verify that ${\mathscr{D}}_{j}$ is indeed a GHD of width ≤ *k* of *H*_*j*_.

1. Consider an arbitrary *f* ∈ *E*(*H*_*j*_). We have to show that there exists a node $\hat {n}$ in *T*_*j*_ with $f \subseteq \chi _{j}(\hat {n})$. By the second observation above, we know that *f* ∈ *D*_*i*_ for some *i* ≥ 0. If *f* ∈ *D*_0_, then $f \subseteq \chi _{j}(n)$ for the root node *n* holds and we are done.
  On the other hand, if *f* ∈ *D*_*i*_ for some *i* ≥ 1, then there exists a node $\hat {n}$ in the subtree of $T^{\prime }$ rooted at *n*_*i*_ with $f \subseteq \chi ^{\prime }(\hat {n})$. Moreover, since *D*_*i*_ ∩ *D*_0_ = *∅*, we know that *f*≠*e* and, therefore, *f* ∈ *C*_*j*_ holds. By *f* ∈ *C*_*j*_ and *f* ∈ *D*_*i*_, we have $D_{i} \subseteq C_{j}$. Hence, by our construction of ${\mathscr{D}}_{j}$, $\hat {n}$ is a node in *T*_*j*_. Moreover, $f \subseteq V(H_{j})$ and $f \subseteq \chi ^{\prime }(\hat {n})$. Hence, we also have $f \subseteq \chi _{j}(\hat {n}) = \chi ^{\prime }(\hat {n}) \cap V(H_{j})$.
2. Consider an arbitrary vertex *v* ∈ *V* (*H*_*j*_). We have to show that $\{ \hat {n} \in N_{j} \mid v \in \chi _{j}(\hat {n}) \}$ is a connected subtree of *T*_*j*_, where *N*_*j*_ denotes the node set of *T*_*j*_. Let $\hat {n}_{1}$ and $\hat {n}_{2}$ be two nodes in *N*_*j*_ with $v \in \chi _{j}(\hat {n}_{1})$ and $v \in \chi _{j}(\hat {n}_{2})$. Then also $v \in \chi ^{\prime }(\hat {n}_{1})$ and $v \in \chi ^{\prime }(\hat {n}_{2})$ hold. Hence, in the GHD ${\mathscr{D}}^{\prime }$, for every node $\hat {n}$ on the path between $\hat {n}_{1}$ and $\hat {n}_{2}$, we have $v\in \chi ^{\prime }(\hat {n})$. Hence, every such node $\hat {n}$ also satisfies $v\in \chi _{j}(\hat {n})$ by the definition $\chi _{j}(\hat {n}) = \chi ^{\prime }(\hat {n}) \cap V(H_{j})$.
3. Consider an arbitrary node $\hat {n}$ in *T*_*j*_. We have to show that $\chi _{j}(\hat {n}) \subseteq B(\lambda _{j}(\hat {n}))$ holds. We distinguish the two cases from the definition of $\lambda _{j}(\hat {n})$:
  If $\lambda ^{\prime }(\hat {n}) \subseteq E(H_{j})$ holds, then we have $\lambda _{j}(\hat {n}) = \lambda ^{\prime }(\hat {n})$. Hence, from the property $\chi ^{\prime }(\hat {n}) \subseteq B(\lambda ^{\prime }(\hat {n}))$ for the GHD ${\mathscr{D}}^{\prime }$ and $\chi _{j}(\hat {n}) \subseteq \chi ^{\prime }(\hat {n})$ it follows immediately that $\chi _{j}(\hat {n}) \subseteq B(\lambda _{j}(\hat {n}))$ holds.
  Now suppose that $\lambda ^{\prime }(\hat {n}) \not \subseteq E(H_{j})$ holds and let $\delta = \lambda ^{\prime }(\hat {n}) \setminus E(H_{j}) \neq \emptyset$. In this case, we have $\lambda _{j}(\hat {n}) = (\lambda ^{\prime }(\hat {n}) \setminus \delta ) \cup \{e \}$. By $\chi _{j}(\hat {n}) \subseteq V(H_{j})$, in order to prove $\chi _{j}(\hat {n}) \subseteq B(\lambda _{j}(\hat {n}))$, it suffices to show that $B(\lambda _{j}(\hat {n})) \supseteq B(\lambda ^{\prime }(\hat {n})) \cap V(H_{j})$. To this end, it actually suffices to show that every $f^{\prime } \in \delta$ has the property $f^{\prime } \cap V(H_{j}) \subseteq e$:
    By $f^{\prime } \in \delta$, we have $f^{\prime } \in C_{j^{\prime }}$ for some $j^{\prime } \neq j$. Hence, for every *f* ∈ *C*_*j*_, we have $f^{\prime } \cap f \subseteq e$ by the definition of [*e*]-components. Moreover, of course, also $f^{\prime } \cap e \subseteq e$ holds. Hence, we indeed have $f^{\prime } \cap V(H_{j}) \subseteq e$.
4. Finally, the width of ${\mathscr{D}}_{j}$ is clearly ≤ *k* since $\lambda _{j} (\hat {n})$ is either equal to $\lambda ^{\prime }(\hat {n})$ or we add *e* but only after subtracting a non-empty set *δ* from $\lambda ^{\prime }(\hat {n})$.

⇐: For $j \in \{1, \dots , \ell \}$, let ${\mathscr{D}}_{j} = \langle T_{j}, \chi _{j}, \lambda _{j} \rangle$ be a GHD of *H*_*j*_ of width ≤ *k*. By the definition of GHDs and by the fact that *e* ∈ *E*(*H*_*j*_) holds for every *j*, there exists a node *n*_*j*_ in *T*_*j*_ with $e \subseteq \chi _{j}(n_{j})$. W.l.o.g., we may assume that *n*_*j*_ is the root of *T*_*j*_. Then we construct GHD ${\mathscr{D}} = \langle T, \chi , \lambda \rangle$ as follows:

- The tree structure *T* is obtained by introducing a new node *n* as the root of *T*, whose child nodes are $n_{1}, \dots , n_{j}$ and each tree *T*_*j*_ becomes the subtree of *T* rooted at *n*_*j*_.
- For the root node *n*, we set *χ*(*n*) = *e* and *λ*(*n*) = {*e*}.
- For any other node $\hat {n}$ of *T*, we have that $\hat {n}$ comes from exactly one of the trees *T*_*j*_. We thus set $\chi (\hat {n}) = \chi _{j}(\hat {n})$ and $\lambda (\hat {n}) = \lambda _{j}(\hat {n})$.

Clearly, ${{\mathscr{D}}}$ can be constructed in polynomial time from the GHDs ${\mathscr{D}}_{1}, \dots , {\mathscr{D}}_{\ell }$. Moreover, the width of ${\mathscr{D}}$ is obviously bounded by the maximum width over the GHDs ${\mathscr{D}}_{i}$. It remains to verify that ${{\mathscr{D}}}$ is indeed a GHD of *H*.

1. Consider an arbitrary *f* ∈ *E*(*H*). We have to show that there is a node $\hat {n}$ in *T*, s.t. $f \subseteq \chi (\hat {n})$. By the definition of [*e*]-components, we either have *f* ∈ *C*_*i*_ for some *i* or $f \subseteq e$. If *f* ∈ *C*_*i*_, then there exists a node $\hat {n}$ in the subtree rooted at *n*_*i*_ with $\chi (\hat {n}) = \chi _{i}(\hat {n}) \supseteq f$. If $f \subseteq e$, then we have $f \subseteq \chi (n)$.
2. Consider an arbitrary vertex *v* ∈ *V* (*H*). We have to show that $\{ \hat {n} \in N \mid v \in \chi (\hat {n}) \}$ is a connected subtree of *T*, where *N* denotes the node set of *T*. Let $v \in \chi (\hat {n}_{1})$ and $v \in \chi (\hat {n}_{2})$ for two nodes $\hat {n}_{1}$ and $\hat {n}_{2}$ in *N* and let $\hat {n}$ be on the path between $\hat {n}_{1}$ and $\hat {n}_{2}$. If both nodes are in some subtree *T*_*i*_ of *T*, then the connectedness condition carries over from ${\mathscr{D}}_{i}$ to ${\mathscr{D}}$. If one of the nodes $\hat {n}_{1}$ and $\hat {n}_{2}$ is the root *n* of *T*, say $n = \hat {n}_{1}$, then *v* ∈ *e*. Moreover, we have $e \subseteq \chi (n_{i})$ by our construction of ${\mathscr{D}}$. Hence, we may again use the connectedness condition on ${\mathscr{D}}_{i}$ to conclude that $v \in \chi (\hat {n})$ for every node $\hat {n}$ along the path between $\hat {n}_{1}$ and $\hat {n}_{2}$. Finally, suppose that $\hat {n}_{1}$ and $\hat {n}_{2}$ are in different subtrees *T*_*i*_ and *T*_*j*_. Then *v* ∈ *V* (*H*_*i*_) ∩ *V* (*H*_*j*_) holds and, therefore, *v* ∈ *e* by the construction of *H*_*i*_ and *H*_*j*_ via different [*e*]-components. Hence, we are essentially back to the previous case. That is, we have $v \in \chi (\hat {n})$ for every node $\hat {n}$ along the path from *n* to $\hat {n}_{1}$ and for every node $\hat {n}$ along the path from *n* to $\hat {n}_{2}$. Together with *v* ∈ *χ*(*n*), we may thus conclude that $v \in \chi (\hat {n})$ indeed holds for every node $\hat {n}$ along the path between $\hat {n}_{1}$ and $\hat {n}_{2}$.
3. Consider an arbitrary node $\hat {n}$ in *T*. We have to show that $\chi (\hat {n}) \subseteq B(\lambda (\hat {n}))$. Clearly, all nodes in a subtree *T*_*i*_ inherit this property from the GHD ${\mathscr{D}}_{i}$ and also the root node *n* satisfies this condition by our definition of *χ*(*n*) and *λ*(*n*).

### Completion of the proof of theorem 2

We now make a case distinction over all possible pairs (*i*,*j*) of Rules *i* and *j* applicable to some hypergraphs $H_{i}, H_{j} \in {{\mathscr{H}}}$ and exhibit a concrete hypergraph set ${{\mathscr{H}}}^{\prime }$ that can be obtained from ${{\mathscr{H}}}$ no matter if we first apply Rule *i* to *H*_*i*_ or Rule *j* to *H*_*j*_. Note that we only need to consider the cases *i* ≤ *j*, since the cases *i* > *j* are thus covered by symmetry. Moreover, the only non-trivial case is that both Rules *i* and *j* are applied to the same hypergraph, i.e., *H*_*i*_ = *H*_*j*_ = *H* for some hypergraph $H \in {{\mathscr{H}}}$.

“(i,5)’: local confluence is immediate for any combination of Rule 5 with another rule. Let $H \in {{\mathscr{H}}}$ with $\lvert {E(H)}\rvert \leq k$ and suppose that some other rule is also applicable to *H*. Then the desired hypergraph set ${{\mathscr{H}}}^{\prime }$ is ${{\mathscr{H}}}^{\prime } = {{\mathscr{H}}} \setminus \{H\}$. Clearly, ${{\mathscr{H}}}^{\prime }$ is the result of applying Rule 5 to $H \in {{\mathscr{H}}}$ and no further rule application is required in this case. Now suppose that another rule is applied first to *H*: Rules 1,2, and 3 allow us to delete a vertex or an edge. In particular, the number of edges of the resulting hypergraph is still ≤ *k* and we may apply Rule 5 afterwards to get ${{\mathscr{H}}}^{\prime }$. Now suppose that Rule 4 is applicable to *H*. This means that we may replace *H* by several hypergraphs $H_{1}, \dots , H_{\ell }$ with *ℓ* ≥ 2. However, all these hypergraphs satisfy $\lvert {E(H_{i})}\rvert < \lvert {E(H)}\rvert \leq k$. Hence, we may apply Rule 5 to each of them and delete all of the hypergraphs $H_{1}, \dots , H_{\ell }$ so that we again end up with ${{\mathscr{H}}}^{\prime }$.

“(1,1)”: Suppose that two applications of Rule 1 to some hypergraph $H \in {{\mathscr{H}}}$ are possible. That is, *H* contains a vertex *v*_1_ that only occurs in a single edge *e*_1_ and a vertex *v*_2_ that only occurs in a single edge *e*_2_ with *v*_1_≠*v*_2_. Note that, after deleting *v*_1_ from *V* (*H*), *v*_2_ still occurs in a single edge *e*_2_. Likewise, after deleting *v*_2_ from *V* (*H*), *v*_1_ still occurs in a single edge *e*_1_. Hence, ${{\mathscr{H}}}^{\prime }$ is obtained by replacing *H* in ${{\mathscr{H}}}$ by $H^{\prime }$, which results from deleting both *v*_1_ and *v*_2_ from *V* (*H*).

“(1,2)”: Suppose that an application of Rule 1 and an application of Rule 2 to the same hypergraph $H \in {{\mathscr{H}}}$ are possible. That is, *H* contains a vertex *v* that only occurs in a single edge *e* and *H* contains edges *e*_1_,*e*_2_ with $e_{1} \subseteq e_{2}$. Hence, on one hand, we may delete *v* from *H* by Rule 1 and, on the other hand, we may delete *e*_1_ from *H* by Rule 2. Note that *e*_1_≠*e*, i.e., *v* cannot occur in *e*_1_ since we are assuming that *v* occurs in a single edge and $e_{1} \subseteq e_{2}$. Hence, after deleting *v* from *V* (*H*), deletion of *e*_1_ via Rule 2 is still possible, since we still have $e_{1} \subseteq e_{2}$ and also $e_{1} \subseteq (e_{2} \setminus \{v\})$ (the latter relationship is relevant if *e* = *e*_2_ and we actually delete *v* from *e*_2_). Likewise, *v* still occurs in a single edge *e* after deleting *e*_1_ via Rule 2. Hence, ${{\mathscr{H}}}^{\prime }$ is obtained by replacing *H* in ${{\mathscr{H}}}$ by $H^{\prime }$, which results from deleting both *v* from *V* (*H*) and *e*_1_ from *E*(*H*).

“(1,3)”: Suppose that an application of Rule 1 and an application of Rule 3 to the same hypergraph $H \in {{\mathscr{H}}}$ are possible. That is, *H* contains a vertex *v* that only occurs in a single edge *e* and *H* contains vertices *v*_1_,*v*_2_ of the same type, i.e., they occur in the same edges. If *v* is different from *v*_1_ and *v*_2_, then we transform *H* into $H^{\prime }$ by deleting *v* and *v*_2_ from *H*. If *v* = *v*_2_, then Rule 1 and Rule 3 are simply two different ways of deleting node *v* from *V* (*H*). Hence, the only interesting case remaining is that *v* = *v*_1_ holds. In this case, also *v*_2_ occurs in edge *e* only, since we are assuming that *v*_1_,*v*_2_ are of the same type. Hence, ${{\mathscr{H}}}^{\prime }$ is obtained by replacing *H* by the hypergraph $H^{\prime }$ which results from deleting both *v*_1_ and *v*_2_ from *V* (*H*): if we first delete *v*_1_ via Rule 1 then we may delete *v*_2_ afterwards also via Rule 1. Conversely, if we first delete *v*_2_ via Rule 3, then Rule 1 is still applicable to *v*_1_ and we may thus delete it afterwards.

“(1,4)”: Suppose that an application of Rule 1 and an application of Rule 4 to the same hypergraph $H \in {{\mathscr{H}}}$ are possible. That is, *H* contains a vertex *v*_1_ that only occurs in a single edge *e*_1_ and *H* contains an edge *e* such that *H* has [*e*]-components ${{\mathscr{C}}} = \{C_{1}, \dots , C_{\ell }\}$ with *ℓ* ≥ 2. Let $e_{1}^{\prime } = e_{1} \setminus \{v_{1}\}$.Case 1. Suppose *e*≠*e*_1_. We have $e_{1} \not \subseteq e$ since *v*_1_ only occurs in *e*_1_. Hence, *e*_1_ is contained in some [*e*]-component *C*_*i*_. We distinguish two subcases.Case 1.1. Suppose that (*e*_1_ ∖ *e*) = {*v*_1_}. We are assuming that *v*_1_ only occurs in *e*_1_. Hence, *e*_1_ is not [*e*]-connected with any other edge and we, therefore, have *C*_*i*_ = {*e*_*i*_}. In this case, ${{\mathscr{H}}}^{\prime }$ is obtained by replacing *H* in ${{\mathscr{H}}}$ by the hypergraphs $H_{1}, \dots , H_{i-1}$, $H_{i+1}, \dots , H_{\ell }$ with *E*(*H*_*j*_) = *C*_*j*_ ∪{*e*} for *j*≠*i*. If we first apply Rule 4 to *H*, then we get *ℓ* hypergraphs $H_{1}, \dots , H_{\ell }$ with *E*(*H*_*i*_) = *C*_*i*_ ∪{*e*_1_} = {*e*,*e*_1_}. We may thus delete *e* from *H*_*i*_ by Rule 2 (since we have $e \subseteq e_{1}$) to get $H^{\prime }_{i}$ and then delete $H^{\prime }_{i}$ altogether by Rule 5 (since we have $\lvert {E(H^{\prime }_{i})}\rvert = 1 \leq k$ for any *k* ≥ 1). Conversely, if we first apply Rule 1 and thus delete *v*_1_ from *e*_1_, then *e* and *e*_1_ coincide. Hence, the resulting hypergraph only has *ℓ* − 1 [*e*]-components $C_{1}, \dots , C_{i-1}, C_{i+1}, \dots , C_{\ell }$. Rule 4 therefore allows us to replace this hypergraph by $H_{1}, \dots , H_{i-1}, H_{i+1}, \dots , H_{\ell }$ with *E*(*H*_*j*_) = *C*_*j*_ ∪{*e*} for *j*≠*i*.Case 1.2. Suppose that $(e_{1} \setminus e) \supset \{v_{1}\}$. Moreover, since *v*_1_ occurs in no other edge, *e*_1_ is connected to the other edges in *C*_*i*_ via vertices different from *e*. Hence, after deleting *v*_1_ from *e*_1_, *H* still has *ℓ* [*e*]-components ${{\mathscr{C}}}^{\prime } = \{C_{1}, \dots , C_{i-1}, C^{\prime }_{i}, C_{i+1}$, …, *C*_*ℓ*_} where $C^{\prime }_{i} = (C_{i} \setminus {e_{1}}) \cup \{ e^{\prime }_{1}\}$. In this case, ${{\mathscr{H}}}^{\prime }$ is obtained by replacing *H* in ${{\mathscr{H}}}$ by the hypergraphs $H_{1}, \dots , H_{i-1}, H^{\prime }_{i}, H_{i+1}, \dots$, *H*_*ℓ*_ with $E(H^{\prime }_{i}) = C^{\prime }_{i} \cup \{e\}$ and *E*(*H*_*j*_) = *C*_*j*_ ∪{*e*} for *j*≠*i*. We can get these hypergraphs by first applying Rule 4 to get the hypergraphs $H_{1}, \dots , H_{i-1}, H_{i}$, *H*_*i*+ 1_, $\dots , H_{\ell }$ with *E*(*H*_*i*_) = *C*_*i*_ ∪{*e*} and, afterwards, transforming *H*_*i*_ into $H^{\prime }_{i}$ via Rule 1. Alternatively, we can get these hypergraphs by first replacing *e*_1_ by $e^{\prime }_{1}$ in *H* via Rule 1 and then applying Rule 4 to get the hypergraphs $H_{1}, \dots , H_{i-1}, H^{\prime }_{i}, H_{i+1}, \dots , H_{\ell }$ via the [*e*]-components ${{\mathscr{C}}}^{\prime } = \{C_{1}, \dots , C_{i-1}, C^{\prime }_{i}, C_{i+1}, \dots , C_{\ell }\}$.Case 2. Now suppose *e* = *e*_1_. Let $H^{\prime }$ with $E(H^{\prime }) = (E(H) \setminus \{e_{1}\}) \cup \{e^{\prime }_{1}\}$. Since *v*_1_ only occurs in *e*_1_, there is no difference between the [*e*_1_]-components of *H* and the $[e^{\prime }_{1}]$-components of $H^{\prime }$. Hence, in this case, ${{\mathscr{H}}}^{\prime }$ is obtained by replacing *H* in ${{\mathscr{H}}}$ by the hypergraphs $H^{\prime }_{1}, \dots , H^{\prime }_{\ell }$ with $E(H^{\prime }_{i}) = C_{i} \cup \{e^{\prime }_{1}\}$ for every $i \in \{1, \dots , \ell \}$. We can get these hypergraphs by first deleting *v*_1_ from *e*_1_ via Rule 1 to get hypergraph $H^{\prime }$ and then applying Rule 4 to $H^{\prime }$, where the $[e^{\prime }_{1}]$-components of $H^{\prime }$ are precisely ${{\mathscr{C}}} = \{C_{1}, \dots , C_{\ell }\}$. Or we may first apply Rule 4 to *H* to get the hypergraphs $H_{1}, \dots , H_{\ell }$ with *E*(*H*_*i*_) = *C*_*i*_ ∪{*e*} with *e* = *e*_1_ and then apply Rule 1 to each of the resulting hypergraphs *H*_*i*_ and replace *e*_1_ by $e^{\prime }_{1}$ in each of them.

“(2,2)”: Suppose that two applications of Rule 2 to the same hypergraph ${H \in {\mathscr{H}}}$ are possible. That is, *H* contains edges $e_{1}, e^{\prime }_{1}$, such that $e_{1} \subseteq e^{\prime }_{1}$ and edges $e_{2}, e^{\prime }_{2}$, such that $e_{2} \subseteq e^{\prime }_{2}$. Then ${{\mathscr{H}}}^{\prime }$ is obtained by replacing *H* in ${{\mathscr{H}}}$ by $H^{\prime }$ such that $E(H^{\prime }) = E(H) \setminus \{e_{1},e_{2}\}$. If $e^{\prime }_{1} \neq e_{2}$ and $e^{\prime }_{2} \neq e_{1}$, then it makes no difference whether we first delete *e*_1_ or *e*_2_. In either case, we may afterwards delete the other edge via Rule 2.

Now suppose that $e^{\prime }_{1} = e_{2}$ holds. The case $e^{\prime }_{2} = e_{1}$ is symmetric. Then, by $e_{2} \subseteq e^{\prime }_{2}$, we also have $e_{1} \subseteq e^{\prime }_{2}$. Hence, Rule 2 is applicable to $e_{1},e^{\prime }_{2}$ (thus allowing us to delete *e*_1_) and also to $e_{2},e^{\prime }_{2}$ (thus allowing us to delete *e*_2_). Hence, again, no matter whether we first delete *e*_1_ or *e*_2_, we are afterwards allowed to delete also the other edge via Rule 2.

“(2,3)”: Suppose that an application of Rule 2 and an application of Rule 3 to the same hypergraph $H \in {{\mathscr{H}}}$ are possible. That is, *H* contains edges *e*_1_,*e*_2_, such that $e_{1} \subseteq e_{2}$ and vertices *v*_1_,*v*_2_ of the same type. Hence, on one hand, we may delete *e*_1_ from *H* by Rule 2 and, on the other hand, we may delete *v*_2_ from *H* by Rule 3.

First, suppose that *v*_2_∉*e*_1_. Then also *v*_1_∉*e*_1_. Hence, after deleting *v*_2_ from *H* via Rule 3, the resulting hypergraph still contains edges $e_{1},e^{\prime }_{2}$ with $e_{1} \subseteq e^{\prime }_{2}$, where $e^{\prime }_{2} = e_{2}$ (if *v*_2_∉*e*_2_) or $e^{\prime }_{2} = e_{2} \setminus \{v_{2}\}$ (if *v*_2_ ∈ *e*_2_). Hence, after deleting *v*_2_ from *H* via Rule 3, we may still delete *e*_1_ via Rule 2. Conversely, if we first delete *e*_1_ from *H*, then *v*_1_ and *v*_2_ still have the same type and we may delete *v*_2_ afterwards.

It remains to consider the case *v*_2_ ∈ *e*_1_. Then also *v*_2_ ∈ *e*_2_. Hence, after deleting *v*_2_ from *H* via Rule 3, the resulting hypergraph contains the edges $e^{\prime }_{1} = e_{1} \setminus \{v_{2}\}$ and $e^{\prime }_{2} = e_{2} \setminus \{v_{2}\}$ with $e^{\prime }_{1} \subseteq e^{\prime }_{2}$. Hence, after deleting *v*_2_ from *H* via Rule 3, we may delete $e^{\prime }_{1}$ via Rule 2. Conversely, if we first delete *e*_1_ from *H*, then *v*_1_ and *v*_2_ still have the same type and we may delete *v*_2_ afterwards.

“(2,4)”: Suppose that an application of Rule 2 and an application of Rule 4 to the same hypergraph $H \in {{\mathscr{H}}}$ are possible. That is, *H* contains edges *e*_1_,*e*_2_, such that $e_{1} \subseteq e_{2}$ and *H* contains an edge *e* such that *H* has [*e*]-components ${{\mathscr{C}}} = \{C_{1}, \dots , C_{\ell }\}$ with *ℓ* ≥ 2. We distinguish several cases and subcases:Case 1. Suppose that *e*_1_≠*e*.Case 1.1. If $e_{1} \subseteq e$, then ${{\mathscr{H}}}^{\prime }$ is obtained by replacing *H* in ${{\mathscr{H}}}$ by $H_{1}, \dots , H_{\ell }$ with *E*(*H*_*i*_) = *C*_*i*_ ∪{*e*} for every $i \in \{1, \dots , \ell \}$. If we first apply Rule 4 to *H*, then the subedges of *e* are not contained in any of the components *C*_*i*_. Hence, we do not even need to apply Rule 2 anymore to get rid of edge *e*_1_. Alternatively, if we first delete *e*_1_ via Rule 2, then Rule 4 is still applicable to $H^{\prime }$ with $E(H^{\prime }) = E(H) \setminus \{e_{1}\}$, and we get exactly the same hypergraphs $H_{1}, \dots , H_{\ell }$ as before.Case 1.2. If $e_{1} \not \subseteq e$, then also $e_{2} \not \subseteq e$ and both *e*_1_,*e*_2_ are contained in exactly one [*e*]-component *C*_*i*_. In this case, ${{\mathscr{H}}}^{\prime }$ is obtained by replacing *H* in ${{\mathscr{H}}}$ by $H_{1}, \dots , H_{i-1},H^{\prime }_{i}$, $H_{i+1}, {\dots } H_{\ell }$ with $E(H^{\prime }_{i}) = (C_{i} \setminus \{e_{1}\}) \cup \{e\}$ and *E*(*H*_*j*_) = *C*_*j*_ ∪{*e*} for *j*≠*i*. If we first apply Rule 4 to *H*, then we get the hypergraphs $H_{1}, \dots , H_{i-1},H_{i}, H_{i+1}, {\dots } H_{\ell }$ with *H*_*i*_ = *C*_*i*_ ∪{*e*}. Now Rule 2 is applicable to *H*_*i*_ and we may delete *e*_1_ from *H*_*i*_ to get $H^{\prime }_{i}$. Conversely, we may first apply Rule 2 to delete *e*_1_ from *H*. Let $H^{\prime }$ with $E(H^{\prime }) = E(H) \setminus \{e_{1}\}$ denote the resulting hypergraph. Then $H^{\prime }$ has the [*e*]-components ${{\mathscr{C}}}^{\prime } = \{C_{1}, \dots , C_{i-1}, C^{\prime }_{i},C_{i+1}, \dots , C_{\ell }\}$ with *ℓ* ≥ 2 and $C^{\prime }_{i} = C_{i} \setminus \{e_{1}\}$. Note that $C^{\prime }_{i} \neq \emptyset$, since *e*_2_ ∈ *C*_*i*_. Hence, application of Rule 4 to $H^{\prime }$ yields the same hypergraphs $H_{1}, \dots , H_{i-1}$, $H^{\prime }_{i}, H_{i+1}, {\dots } H_{\ell }$ as before.Case 2. Suppose that *e*_1_ = *e*. Then *e*_2_ is contained in one of the [*e*]-components. W.l.o.g., assume *e*_2_ ∈ *C*_*ℓ*_. Now let ${{\mathscr{D}}} = \{D_{1}, \dots , D_{m}\}$ denote the [*e*_2_]-components of *H*. By $e \subseteq e_{2}$, every [*e*_2_]-component *D*_*j*_ is contained in exactly one [*e*]-component *C*_*i*_. That is, every [*e*_2_]-connected set of edges of *E*(*H*) is also [*e*]-connected but the converse is, in general, not true. Such a situation that the converse is not true may happen if a path connecting two edges uses one of the vertices in *e*_2_ ∖ *e*. Note however that only the [*e*]-component *C*_*ℓ*_ with *e*_2_ ∈ *C*_*ℓ*_ contains vertices in *e*_2_ ∖ *e*. Hence, the [*e*]-components $C_{1}, \dots , C_{\ell -1}$ are also [*e*_2_]-components and we may set *D*_*i*_ = *C*_*i*_ for every $i \in \{1, \dots , \ell -1\}$. For the [*e*]-component *C*_*ℓ*_, we distinguish the following 2 subcases:Case 2.1. If all edges in *C*_*ℓ*_ are subedges of *e*_2_, then the [*e*_2_]-components of *H* are ${{\mathscr{D}}} = \{D_{1}, \dots , D_{\ell -1} \}$. In this case, we obtain ${{\mathscr{H}}}^{\prime }$ by replacing *H* in ${{\mathscr{H}}}$ by $H_{1}, \dots , H_{\ell -1}$ with *E*(*H*_*i*_) = *C*_*i*_ ∪{*e*} for every $i \in \{1, \dots , \ell -1\}$. If we first apply Rule 4 to *H*, then we get the hypergraphs $H_{1}, \dots , H_{\ell -1}, H_{\ell }$ with *E*(*H*_*ℓ*_) = *C*_*ℓ*_ ∪{*e*}. Since we are assuming that *e*_2_ ∈ *C*_*ℓ*_ and all edges in *C*_*ℓ*_ are subedges of *e*_2_, we may apply Rule 2 to *H*_*ℓ*_ multiple times to delete all edges except for *e*_2_. Finally, when *H*_*ℓ*_ has been reduced to a hypergraph consisting of a single edge, we may delete *H*_*ℓ*_ altogether by Rule 5.

Conversely, we may first delete *e* from *H* via Rule 2. That is, we get hypergraph $H^{\prime }$ with $E(H^{\prime }) = E(H) \setminus \{e\}$. Then the [*e*_2_]-components of $H^{\prime }$ are simply ${{\mathscr{D}}} = \{D_{1}, \dots , D_{\ell -1} \}$, i.e., the subedge *e* ∈ *e*_2_ is not contained in any of the [*e*_2_]-components of *H* anyway.Case 2.1.1. If *ℓ* ≥ 3, then we may apply Rule 4 to $H^{\prime }$ and replace $H^{\prime }$ by $H^{\prime }_{1}, \dots , H^{\prime }_{\ell -1}$ with $H^{\prime }_{i} = D_{i} \cup \{e_{2}\}$. Recall that *C*_*i*_ = *D*_*i*_ for every $i \in \{1, \dots , \ell -1\}$ and that none of the vertices in *e*_2_ ∖ *e* occurs in *C*_*i*_. Hence, each $H^{\prime }_{i}$ is actually of the form $H^{\prime }_{i} = C_{i} \cup \{e_{2}\}$. Moreover, in each $H^{\prime }_{i}$, the vertices in *e*_2_ ∖ *e* only occur in *e*_2_ and nowhere else in $H^{\prime }_{i}$. Hence, in every hypergraph $H^{\prime }_{i}$, we may delete each of the vertices in *e*_2_ ∖ *e* via Rule 1 so that we ultimately reduce *e*_2_ to *e*. That is, we transform every $H^{\prime }_{i}$ into *H*_*i*_ and we thus indeed replace *H* by $H_{1}, \dots , H_{\ell -1}$.Case 2.1.2. If *ℓ* = 2, then *H* and also $H^{\prime }$ consists of a single [*e*_2_]-component *D*_1_ = *C*_1_. Moreover, all edges in $E(H^{\prime }) \setminus D_{1}$ are subedges of *e*_2_. Hence, $E(H^{\prime }) \setminus D_{1}$ is of the form $\{e_{2}, f_{1}, \dots , f_{m}\}$ with *m* ≥ 0, such that $f_{j} \subseteq e_{2}$ holds for every *j*. Hence, we may delete all subedges *f*_*j*_ of *e*_2_ via Rule 2 to transform $H^{\prime }$ into *D*_1_ ∪{*e*_2_} = *C*_1_ ∪{*e*_2_}. Then we again have the situation that all vertices in *e*_2_ ∖ *e* only occur in *e*_2_. Hence, we may delete all these vertices via multiple applications of Rule 1. In total, we may thus replace *H* by *H*_1_ with *E*(*H*_1_) = *C*_1_ ∪{*e*}.Case 2.2. If not all edges in *C*_*ℓ*_ are subedges of *e*_2_, then *C*_*ℓ*_ has at least one [*e*_2_]-component. In total, the [*e*_2_]-components of *H* are ${{\mathscr{D}}} = \{D_{1}, \dots , D_{\ell -1}, D_{\ell }, \dots$, *D*_*m*_} with *m* ≥ *ℓ*, such that $\{D_{\ell }, \dots , D_{m}\}$ are the [*e*_2_]-components of *C*_*ℓ*_. In this case, we obtain ${{\mathscr{H}}}^{\prime }$ by replacing *H* in ${{\mathscr{H}}}$ by $H_{1}, \dots , H_{\ell -1},H^{\prime }_{\ell }, \dots , H^{\prime }_{m}$ with *E*(*H*_*i*_) = *C*_*i*_ ∪{*e*} for every $i \in \{1, \dots , \ell -1\}$ and $E(H^{\prime }_{j}) = D_{j} \cup \{e_{2}\}$ for every $j \in \{\ell , \dots , m\}$. If we first apply Rule 4 (w.r.t. to edge *e*) to *H*, then we get the hypergraphs $H_{1}, \dots , H_{\ell -1}, H_{\ell }$ with *E*(*H*_*ℓ*_) = *C*_*ℓ*_ ∪{*e*}. Now consider *H*_*ℓ*_.Case 2.2.1. If *H*_*ℓ*_ consists of a single [*e*_2_]-component *D*_*ℓ*_, then we simply delete all edges in *E*(*H*_*ℓ*_) ∖ *D*_*ℓ*_ to get $H^{\prime }_{\ell } = D_{\ell } \cup \{e_{2}\}$. This is possible since all edges in *E*(*H*_*ℓ*_) ∖ *D*_*ℓ*_ are subedges of *e*_2_ and we may therefore delete them via Rule 2. Conversely, suppose that we first delete *e* from *H* via Rule 2 to get $H^{\prime }$ with $E(H^{\prime }) = E(H) \setminus \{e\}$. Then we may apply Rule 4 (w.r.t. edge *e*_2_) and replace $H^{\prime }$ by $H^{\prime }_{1}, \dots , H^{\prime }_{\ell }$ with $E(H^{\prime }_{i}) = D_{i} \cup \{e_{2}\}$ for every $i \in \{1, \dots , \ell \}$. Again, for $i \in \{1, \dots , \ell -1\}$, we have *D*_*i*_ = *C*_*i*_ and the vertices in *e*_2_ ∖ *e* do not occur in *C*_*i*_. Hence, in each hypergraph $H^{\prime }_{i}$ with $i \in \{1, \dots , \ell -1\}$ we may delete all vertices in *e*_2_ ∖ *e* by multiple applications of Rule 1. In total, we thus replace *H* by $H_{1}, \dots , H_{\ell -1}, H^{\prime }_{\ell }$ as before.Case 2.2.2. If *H*_*ℓ*_ consists of several [*e*_2_]-components $D_{\ell }, \dots , D_{m}$ with *m* > *ℓ*, then we may apply Rule 4 to *H*_*ℓ*_ and replace *H*_*ℓ*_ by $H^{\prime }_{\ell }, \dots , H^{\prime }_{m}$ with $E(H^{\prime }_{j}) = D_{j} \cup \{e_{2}\}$ for every $j \in \{\ell , \dots , m\}$. Conversely, suppose that we first delete *e* from *H* via Rule 2 to get $H^{\prime }$ with $E(H^{\prime }) = E(H) \setminus \{e\}$. Then we may apply Rule 4 (w.r.t. edge *e*_2_) and replace $H^{\prime }$ by $H^{\prime }_{1}, \dots , H^{\prime }_{m}$ with $E(H^{\prime }_{i}) = D_{i} \cup \{e_{2}\}$ for every $i \in \{1, \dots , m\}$. Moreover, as in Case 2.2.1, every $H^{\prime }_{i}$ with $i \in \{1, \dots , \ell - 1\}$ can be transformed into *H*_*i*_ with *E*(*H*_*i*_) = *C*_*i*_ ∪{*e*} by deleting all vertices in *e*_2_ ∖ *e* via multiple applications of Rule 1 and using the equality *C*_*i*_ = *D*_*i*_ for $i \in \{1, \dots , \ell - 1\}$.

“(3,3)”: Suppose that two applications of Rule 3 to the same hypergraph ${H \in {\mathscr{H}}}$ are possible. That is, *H* contains vertices $v_{1}, v^{\prime }_{1}$ of the same type and vertices $v_{2}, v^{\prime }_{2}$ of the same type. Then ${{\mathscr{H}}}^{\prime }$ is obtained by replacing *H* in ${{\mathscr{H}}}$ by $H^{\prime }$ such that *v*_1_ and *v*_2_ are deleted from all edges in *H* and, thus from *V* (*H*) altogether. If $v^{\prime }_{1} \neq v_{2}$ and $v^{\prime }_{2} \neq v_{1}$, then it makes no difference whether we first delete *v*_1_ or *v*_2_. In either case, we may afterwards also delete the other vertex via Rule 3.

Now suppose that $v^{\prime }_{1} = v_{2}$ holds. The case $v^{\prime }_{2} = v_{1}$ is symmetric. Then, all vertices $v_{1}, v^{\prime }_{1},v_{2},v^{\prime }_{2}$ have the same type. Hence, Rule 3 is applicable to $v_{1},v^{\prime }_{2}$ (thus allowing us to delete *v*_1_) and also to $v_{2},v^{\prime }_{2}$ (thus allowing us to delete *v*_2_). Hence, again, no matter whether we first delete *v*_1_ or *v*_2_, we are afterwards allowed to delete also the other vertex via Rule 3.

“(3,4)”: Suppose that an application of Rule 3 and an application of Rule 4 to the same hypergraph $H \in {{\mathscr{H}}}$ are possible. That is, *H* contains vertices *v*_1_,*v*_2_ of the same type and an edge *e* such that *H* has [*e*]-components ${{\mathscr{C}}} = \{C_{1}, \dots , C_{\ell }\}$ with *ℓ* ≥ 2. For any edge *f*, we write $f^{\prime }$ to denote $f^{\prime } = f \setminus \{v_{2}\}$.Case 1. Suppose that *v*_2_∉*e*. Then *v*_2_ is contained in *V* (*C*_*i*_) for precisely one [*e*]-component *C*_*i*_. Moreover, since *v*_1_ has the same type as *v*_2_, also the set $C^{\prime }_{i}$ obtained from *C*_*i*_ by deleting *v*_2_ from all edges remains [*e*]-connected. This is because that all paths that use the vertex *v*_2_ may also use the vertex *v*_1_ instead. Hence, after deleting *v*_2_ from *V* (*H*), *H* still has *ℓ* [*e*]-components ${{\mathscr{C}}}^{\prime } = \{C_{1}, \dots , C_{i-1}, C^{\prime }_{i}$, $C_{i+1}, \dots , C_{\ell }\}$. In this case, ${{\mathscr{H}}}^{\prime }$ is obtained by replacing *H* in ${{\mathscr{H}}}$ by the hypergraphs $H_{1}, \dots , H_{i-1}, H^{\prime }_{i}$, $H_{i+1}, \dots , H_{\ell }$ with $E(H^{\prime }_{i}) = C^{\prime }_{i} \cup \{e\}$ and *E*(*H*_*j*_) = *C*_*j*_ ∪{*e*} for *j*≠*i*. We can thus get these hypergraphs by first applying Rule 4 to get the hypergraphs $H_{1}, \dots , H_{i-1}, H_{i}$, *H*_*i*+ 1_, $\dots , H_{\ell }$ with *E*(*H*_*i*_) = *C*_*i*_ ∪{*e*} and, afterwards, transforming *H*_*i*_ into $H^{\prime }_{i}$ via Rule 3. Alternatively, we can get these hypergraphs by first deleting *v*_2_ from *H* via Rule 3 and then applying Rule 4 to get the hypergraphs $H_{1}, \dots , H_{i-1}, H^{\prime }_{i}, H_{i+1}, \dots , H_{\ell }$ via the [*e*]-components ${{\mathscr{C}}}^{\prime } = \{C_{1}, \dots , C_{i-1}, C^{\prime }_{i}, C_{i+1}, \dots , C_{\ell }\}$.Case 2. Suppose that *v*_2_ ∈ *e*. Let $e^{\prime } = e \setminus \{v_{2}\}$. Suppose that we first transform *H* into $H^{\prime }$ by deleting *v*_2_ from *H* via Rule 3. Then the $[e^{\prime }]$-components of $H^{\prime }$ are ${{\mathscr{C}}}^{\prime } = \{C^{\prime }_{1}, \dots , C^{\prime }_{\ell }\}$ where, for every $i \in \{1, \dots , \ell \}$, $C^{\prime }_{i}$ is obtained from *C*_*i*_ either by deleting *v*_2_ from *V* (*C*_*i*_) if *v*_2_ ∈ *V* (*C*_*i*_) or by setting $C^{\prime }_{i} = C_{i}$ otherwise. Note that here we do not even make use of the fact that *v*_1_ and *v*_2_ have the same type. As long as a vertex *v*_2_ ∈ *e* is deleted from *e* and from all other edges, the [*e*]-components of *H* and the $[e^{\prime }]$-components of $H^{\prime }$ are exactly the same (apart from the fact, of course, that $H^{\prime }$ and, hence, its $[e^{\prime }]$-components no longer contain vertex *v*_2_). We may thus apply Rule 4 to replace $H^{\prime }$ by the set of hypergraphs $\{H^{\prime }_{1}, \dots , H^{\prime }_{\ell }\}$ with $H^{\prime }_{i} = C^{\prime }_{i} \cup \{e^{\prime }\}$. Alternatively, we may first apply Rule 4 to replace *H* by the hypergraphs $H_{1}, \dots , H_{\ell }$ with *E*(*H*_*i*_) = *C*_*i*_ ∪{*e*}. Then, in every *H*_*i*_, we still have the property that *v*_1_ and *v*_2_ have the same type. Hence, we may apply Rule 3 to each hypergraph *H*_*i*_ and delete *v*_2_ from *V* (*H*_*i*_). This results in the same set of hypergraphs $\{H^{\prime }_{1}, \dots , H^{\prime }_{\ell }\}$ as before.

“(4,4)”: Suppose that two applications of Rule 4 to the same hypergraph ${H \in {\mathscr{H}}}$ are possible. That is, *H* contains edges *e*_1_≠*e*_2_, such that *H* has [*e*_1_]-components ${{\mathscr{C}}} = \{C_{1}, \dots , C_{\ell }\}$ with *ℓ* ≥ 2 and *H* has [*e*_2_]-components ${{\mathscr{D}}} = \{D_{1}, \dots , D_{m}\}$ with *m* ≥ 2. Recall that we are assuming that each hypergraph ${H \in {\mathscr{H}}}$ consists of a single connected component.Case 1. Suppose that $e_{1} \subseteq e_{2}$ or $e_{2} \subseteq e_{1}$ holds. The cases are symmetric, so we only need to consider $e_{1} \subseteq e_{2}$. This case is very similar to “(2,4)”, Case 2, where *e*_1_ now plays the role of *e* from “(2,4)”. Indeed, w.l.o.g., we again assume *e*_2_ ∈ *C*_*ℓ*_. If Rule 4 is applied to the [*e*_1_]-components first, then we end up in precisely the same situation as with “(2,4)”. On the other hand, if Rule 4 is applied to the [*e*_2_]-components first, then all subedges of *e*_2_ are actually deleted – including *e*_1_. Hence, we again end up in precisely the same situation as with “(2,4)”.Case 2. Suppose that $e_{1} \not \subseteq e_{2}$ and $e_{2}\not \subseteq e_{1}$ holds. Let *d* = *e*_1_ ∩ *e*_2_.Case 2.1. Suppose that *d* = *∅*. The edge *e*_1_ lies in exactly one [*e*_2_]-component and *e*_2_ lies in exactly one [*e*_1_]-component. W.l.o.g., assume *e*_1_ ∈ *D*_*m*_ and *e*_2_ ∈ *C*_*ℓ*_. We claim that then all of $D_{1} \cup {\dots } \cup D_{m-1} \cup \{e_{2}\}$ is contained in *C*_*ℓ*_. This can be seen as follows: we are assuming that *H* is connected. Then also $D_{1} \cup {\dots } \cup D_{m-1} \cup \{e_{2}\}$ is connected, i.e., there is a path between any two vertices in $D_{1} \cup {\dots } \cup D_{m-1} \cup \{e_{2}\}$ and this path does not need to make use of any edge in *D*_*m*_. This follows immediately from the fact $V(D_{m}) \cap (V(D_{1}) \cup {\dots } \cup V(D_{m-1}) \cup e_{2}) \subseteq e_{2}$, which holds by the definition of components. Moreover, *e*_1_ ∈ *D*_*m*_ and we are assuming *e*_1_ ∩ *e*_2_ = *∅*. Hence, $e_{1} \cap \big (V(D_{1}) \cup {\dots } \cup V(D_{m-1}) \cup e_{2} \big ) = \emptyset$. This means that, if $D_{1} \cup {\dots } \cup D_{m-1} \cup \{e_{2}\}$ is connected, then it is in fact [*e*_1_]-connected, i.e., it lies in a single [*e*_1_]-component, namely *C*_*ℓ*_. By symmetry, also $C_{1} \cup {\dots } \cup C_{\ell -1} \cup \{e_{1}\}$ is contained in a single [*e*_2_]-component, namely *D*_*m*_.

Let ${{\mathscr{H}}}^{\prime }$ be the set of hypergraphs obtained from ${{\mathscr{H}}}$ by replacing *H* in ${{\mathscr{H}}}$ by the following set of hypergraphs: $G_{1}, \dots , G_{\ell -1}, H_{1}, \dots , H_{m-1}, K$ with *E*(*G*_*i*_) = *C*_*i*_ ∪{*e*_1_} for every $i \in \{1, \dots , \ell -1\}$, *E*(*H*_*j*_) = *D*_*j*_ ∪{*e*_2_} for every $j \in \{1, \dots , m-1\}$, and *E*(*K*) = (*C*_*ℓ*_ ∩ *D*_*m*_) ∪{*e*_1_,*e*_2_}. It remains to show that ${{\mathscr{H}}}^{\prime }$ can be reached both, if Rule 4 is applied to the [*e*_1_]-components first and also if Rule 4 is applied to the [*e*_2_]-components first. Actually, ${{\mathscr{H}}}^{\prime }$ is fully symmetric w.r.t. *e*_1_ and *e*_2_. Hence, it suffices to show that we can reach ${{\mathscr{H}}}^{\prime }$ if Rule 4 is applied to the [*e*_1_]-components of *H* first.

The application of Rule 4 to the [*e*_1_]-components of *H* allows us to replace *H* by $G_{1}, \dots , G_{\ell }$ with *E*(*G*_*i*_) = *C*_*i*_ ∪{*e*_1_} for every $i \in \{1, \dots , \ell \}$. Next, we apply Rule 4 to the [*e*_2_]-components of *G*_*ℓ*_. As was observed above, the [*e*_2_]-components $D_{1}, \dots , D_{m-1}$ of *H* are fully contained in *C*_*ℓ*_ and, hence, in *E*(*G*_*ℓ*_). Considering $D_{1}, \dots , D_{m-1}$ as [*e*_2_]-components of *G*_*ℓ*_, the application of Rule 4 gives rise to $H_{1}, \dots , H_{m-1}$ with *E*(*H*_*j*_) = *D*_*j*_ ∪{*e*_2_} for every $j \in \{1, \dots , m-1\}$.

It remains to consider the remaining [*e*_2_]-component *D*_*m*_ of *H*, but now restricted to the hypergraph *G*_*ℓ*_ = *C*_*ℓ*_ ∪{*e*_1_}. Note that it suffices to show that (*C*_*ℓ*_ ∩ *D*_*m*_) ∪{*e*_1_} is [*e*_2_]-connected because, in this case, we would indeed get *K* = (*C*_*ℓ*_ ∩ *D*_*m*_) ∪{*e*_1_,*e*_2_} as the remaining [*e*_2_]-component when applying Rule 4 to *G*_*ℓ*_. Suppose to the contrary that (*C*_*ℓ*_ ∩ *D*_*m*_) ∪{*e*_1_} is not [*e*_2_]-connected, i.e., there exist edges *f*_1_,*f*_2_ ∈ (*C*_*ℓ*_ ∩ *D*_*m*_) ∪{*e*_1_}, such that *f*_1_,*f*_2_ are not [*e*_2_]-connected. We distinguish two cases:Case 2.1.1. One of the edges *f*_1_,*f*_2_ is *e*_1_, say *e*_1_ = *f*_1_. That is *e*_1_ and *f*_2_ are not [*e*_2_]-connected in *G*_*ℓ*_. However, they are in the same [*e*_2_]-component *D*_*m*_ in *H*. This means that there is an [*e*_2_]-path in *H* connecting them. Since this [*e*_2_]-path is not in *C*_*ℓ*_ ∪{*e*_1_}, it must make use of an edge *g* in some [*e*_1_]-component *C*_*i*_ with $i \in \{1, \dots , \ell -1\}$. W.l.o.g., assume that this path was chosen with minimal length. We can traverse this path from *f*_2_ via *g* to *e*_1_. By assuming minimal length, the path from *f*_2_ to *g* does not involve any vertex from *e*_1_. But then *f*_2_ and *g* are [*e*_1_]-connected. This contradicts our assumption that *g* and *f*_2_ lie in different [*e*_1_]-components.Case 2.1.2. Suppose that both edges *f*_1_,*f*_2_ are different from *e*_1_. Again, we have the situation that *f*_1_ and *f*_2_ are not [*e*_2_]-connected in *G*_*ℓ*_, but they are in the same [*e*_2_]-component *D*_*m*_ in *H*. This means that there is an [*e*_2_]-path in *H* connecting them. Since this [*e*_2_]-path is not in *C*_*ℓ*_ ∪{*e*_1_}, it must make use of an edge *g* in some *C*_*i*_ with $i \in \{1, \dots , \ell -1\}$. W.l.o.g., assume that this path was chosen with minimal length. It may possibly involve *e*_1_ but, by the minimality, it uses *e*_1_ at most once. If *e*_1_ is not part of the path then we immediately get a contradiction since there exists an [*e*_1_]-path between any of the edges *f*_*i*_ and edge *g*, where *f*_*i*_ and *g* are assumed to lie in different [*e*_1_]-components. On the other hand, if *e*_1_ is part of this path, then it must be either on the path *f*_1_–*g* or *f*_2_–*g* but not both. By symmetry, we may assume w.l.o.g., that *e*_1_ is on the path *f*_1_–*g*. Then the path *f*_2_–*g* is an [*e*_1_]-path. Again, this contradicts our assumption that *g* and *f*_2_ lie in different [*e*_1_]-components.Case 2.2. Suppose that *d*≠*∅*. Let $R_{1}, \dots , R_{n}$ denote the [*d*]-components of *H*. We have *d* ⊂ *e*_*i*_ for both *i* ∈{1,2}, since, in Case 2, we are assuming $e_{1} \not \subseteq e_{2}$ and $e_{2}\not \subseteq e_{1}$. Hence, *e*_1_ and *e*_2_ are each contained in some [*d*]-component.Case 2.2.1. Suppose that *e*_1_ and *e*_2_ are in two different [*d*]-components. W.l.o.g., we may assume that *e*_1_ ∈ *R*_*n*− 1_ and *e*_2_ ∈ *R*_*n*_. We observe that all [*d*]-components except for *R*_*n*− 1_ are also [*e*_1_]-components. Moreover, the remaining [*e*_1_]-components of *H* are entirely contained in *R*_*n*− 1_ since every [*e*_1_]-component is of course also [*d*]-connected. Let $S_{1}, \dots , S_{\alpha }$ with *α* ≥ 1 denote the [*e*_1_]-components of *H* inside *R*_*n*− 1_. Hence, in total, *H* has the [*e*_1_]-components $R_{1}, \dots , R_{n-2}, R_{n}, S_{1}$, …, *S*_*α*_.

Likewise, all [*d*]-components except for *R*_*n*_ are also [*e*_2_]-components and the remaining [*e*_2_]-components of *H* are entirely contained in *R*_*n*_. Let $T_{1}, \dots , T_{\beta }$ with *β* ≥ 1 denote the [*e*_2_]-components of *H* inside *R*_*n*_. Hence, in total, *H* has the [*e*_2_]-components $R_{1}, \dots , R_{n-1}, T_{1}, \dots , T_{\beta }$.

Let ${{\mathscr{H}}}^{\prime }$ be the set of hypergraphs obtained from ${{\mathscr{H}}}$ by replacing *H* in ${{\mathscr{H}}}$ by the following set of hypergraphs: $F_{1}, \dots , F_{n-2}$, $G_{1}, \dots , G_{\alpha }$, $H_{1}, \dots , H_{\beta }$ with *F*_*i*_ = *R*_*i*_ ∪{*d*} for every $i \in \{1, \dots , n-2\}$, *G*_*i*_ = *S*_*i*_ ∪{*e*_1_} for every $i \in \{1, \dots , \alpha \}$, *H*_*i*_ = *T*_*i*_ ∪{*e*_2_} for every $i \in \{1, \dots , \beta \}$. It remains to show that ${{\mathscr{H}}}^{\prime }$ can be reached both, if Rule 4 is applied to the [*e*_1_]-components first and also if Rule 4 is applied to the [*e*_2_]-components first. Actually, ${{\mathscr{H}}}^{\prime }$ is fully symmetric w.r.t. *e*_1_ and *e*_2_. Hence, it suffices to show that we can reach ${{\mathscr{H}}}^{\prime }$ if Rule 4 is applied to the [*e*_1_]-components of *H* first.

As observed above, the [*e*_1_]-components of *H* are $R_{1}, \dots , R_{n-2}, R_{n}, S_{1}, \dots$, *S*_*α*_. Hence, we may replace *H* by the hypergraphs $F^{\prime }_{1}, \dots , F^{\prime }_{n-2},F^{\prime }_{n}$, $G_{1}, \dots$, *G*_*α*_, where the *G*_*i*_’s are defined as above and the hypergraphs $F^{\prime }_{i}$ with *i*≠*n* − 1 are obtained as $E(F^{\prime }_{i}) = R_{i} \cup \{e_{1}\}$. By assumption, *e*_1_ is in the [*d*]-component *R*_*n*− 1_. Hence, $e_{1} \cap V(R_{i}) \subseteq d$ for all *i*≠*n* − 1. In other words, the vertices in *e*_1_ ∖ *d* only occur in a single edge of $F^{\prime }_{i}$ with *i*≠*n* − 1, namely in the edge *e*_1_. We may therefore apply Rule 1 multiple times to each of the hypergraphs $F^{\prime }_{i}$ with *i*≠*n* − 1. In this way, we replace *e*_1_ in each of these hypergraphs by *d* and we indeed transform $F^{\prime }_{i}$ into *F*_*i*_ for every *i* ≤ *n* − 2.

Also in $F^{\prime }_{n} = R_{n} \cup \{e_{1}\}$ we thus replace *e*_1_ by *d*. Recall that we are assuming that *e*_2_ ∈ *R*_*n*_. Hence, we may delete *d* by Rule 2 since, $d \subseteq e_{2}$. Hence, $F^{\prime }_{n}$ is ultimately transformed into *R*_*n*_. Now consider the [*e*_2_]-components of *H* inside *R*_*n*_, namely $T_{1}, \dots , T_{\beta }$ with *β* ≥ 1. These are also the [*e*_2_]-components of the hypergraph *R*_*n*_, i.e., $T_{i} \subseteq E(R_{n})$ and *T*_*i*_ is (maximally) [*e*_2_]-connected for every *i*. If *β* ≥ 2, then we may apply Rule 4 to *R*_*n*_ and we get precisely the desired hypergraphs *H*_*i*_ = *T*_*i*_ ∪{*e*_2_} for every $i \in \{1, \dots , \beta \}$. On the other hand, if *β* = 1, then *R*_2_ has a single [*e*_2_]-component *T*_1_. Note that all edges of a hypergraph not contained in any of its [*e*_2_]-components are subedges of *e*_2_. Hence, we may again transform *R*_*n*_ into *H*_1_ = *T*_1_ ∪{*e*_2_} by multiple applications of Rule 2, which allows us to delete all subedges of *e*_2_.Case 2.2.2. Suppose that *e*_1_ and *e*_2_ are in the same [*d*]-component. W.l.o.g., we may assume that $\{e_{1},e_{2}\} \subseteq R_{n}$. We observe that all [*d*]-components except for *R*_*n*_ are also [*e*_1_]-components and [*e*_2_]-components. Moreover, the remaining [*e*_1_]-components of *H* and also the remaining [*e*_2_]-components of *H* are entirely contained in *R*_*n*_. Let $S_{1}, \dots , S_{\alpha }$ with *α* ≥ 1 denote the [*e*_1_]-components of *H* inside *R*_*n*_ and let $T_{1}, \dots , T_{\beta }$ with *β* ≥ 1 denote the [*e*_2_]-components of *H* inside *R*_*n*_. Then, in total, *H* has the [*e*_1_]-components $R_{1}, \dots , R_{n-1}, S_{1}$, …, *S*_*α*_ and the [*e*_2_]-components $R_{1}, \dots , R_{n-1}, T_{1}, \dots , T_{\beta }$.

We are assuming that $\{e_{1},e_{2}\} \subseteq R_{n}$. Hence, *e*_1_ is in precisely one [*e*_2_]-component *T*_*j*_ inside *R*_*n*_ and *e*_2_ is in precisely one [*e*_1_]-component *S*_*i*_ inside *R*_*n*_. W.l.o.g., we may assume that *e*_1_ ∈ *T*_*β*_ and *e*_2_ ∈ *S*_*α*_. Analogously to the Case 2.1, we claim that then all of $T_{1} \cup {\dots } \cup T_{\beta -1} \cup \{e_{2}\}$ is contained in *S*_*α*_. This can be seen as follows: we are assuming that *H* is connected. Then also $T_{1} \cup {\dots } \cup T_{\beta -1} \cup \{e_{2}\}$ is connected and even [*e*_1_]-connected, since *e*_1_ ∈ *T*_*β*_. Hence, $T_{1} \cup {\dots } \cup T_{\beta -1} \cup \{e_{2}\}$ lies in a single [*e*_1_]-component, namely *S*_*α*_. By symmetry, also $S_{1} \cup {\dots } \cup S_{\alpha -1} \cup \{e_{1}\}$ is contained in a single [*e*_2_]-component, namely *T*_*β*_.

We now define the set ${{\mathscr{H}}}^{\prime }$ of hypergraphs by combining the ideas of the Cases 2.1 and 2.2.1. Let ${{\mathscr{H}}}^{\prime }$ be the set of hypergraphs obtained from ${{\mathscr{H}}}$ by replacing *H* in ${{\mathscr{H}}}$ by the following set of hypergraphs: $F_{1}, \dots , F_{n-1}$, $G_{1}, \dots , G_{\alpha -1}$, $H_{1}, \dots , H_{\beta -1}$, *K* with *E*(*F*_*i*_) = *R*_*i*_ ∪{*d*} for every $i \in \{1, \dots , n-1\}$, *E*(*G*_*i*_) = *S*_*i*_ ∪{*e*_1_} for every $i \in \{1, \dots , \alpha -1\}$, *E*(*H*_*i*_) = *T*_*i*_ ∪{*e*_2_} for every $i \in \{1, \dots , \beta -1\}$, and *E*(*K*) = (*S*_*α*_ ∩ *T*_*β*_) ∪{*e*_1_,*e*_2_}. It remains to show that ${{\mathscr{H}}}^{\prime }$ can be reached both, if Rule 4 is applied to the [*e*_1_]-components first and also if Rule 4 is applied to the [*e*_2_]-components first. Again, since ${{\mathscr{H}}}^{\prime }$ is fully symmetric w.r.t. *e*_1_ and *e*_2_, it suffices to show that we can reach ${{\mathscr{H}}}^{\prime }$ if Rule 4 is applied to the [*e*_1_]-components of *H* first.

As observed above, the [*e*_1_]-components of *H* are $R_{1}, \dots , R_{n-1}, S_{1}, \dots , S_{\alpha }$. Hence, we may replace *H* in ${{\mathscr{H}}}$ by the hypergraphs $F^{\prime }_{1}, \dots , F^{\prime }_{n-1}$, $G_{1}, \dots , G_{\alpha }$, where the the hypergraphs $F^{\prime }_{i}$ with *i* ≤ *n* − 1 are obtained as $E(F^{\prime }_{i}) = R_{i} \cup \{e_{1}\}$ and *E*(*G*_*α*_) = *S*_*α*_ ∪{*e*_1_}. For $i \in \{1, \dots , \alpha -1\}$, *G*_*i*_ is as defined above, i.e., *E*(*G*_*i*_) = *S*_*i*_ ∪{*e*_1_}. By assumption, *e*_1_ is in the [*d*]-component *R*_*n*_. Hence, $e_{1} \cap V(R_{i}) \subseteq d$ for all *i* ≤ *n* − 1. In other words, the vertices in *e*_1_ ∖ *d* only occur in a single edge of $F^{\prime }_{i}$ with *i* ≤ *n* − 1, namely in the edge *e*_1_. We may therefore apply Rule 1 multiple times to each of the hypergraphs $F^{\prime }_{i}$ with *i* ≤ *n* − 1. In this way, we replace *e*_1_ in each of these hypergraphs by *d* and we indeed transform $F^{\prime }_{i}$ into *F*_*i*_ for every *i* ≤ *n* − 1.

Now consider the hypergraph *G*_*α*_ with *E*(*G*_*α*_) = *S*_*α*_ ∪{*e*_1_}. We apply Rule 4 to the [*e*_2_]-components of *G*_*α*_. As was observed above, the [*e*_2_]-components $T_{1}, \dots , T_{\beta -1}$ of *H* are fully contained in *S*_*α*_ and, hence, in *E*(*G*_*α*_). Considering $T_{1}, \dots , T_{\beta -1}$ as [*e*_2_]-components of *G*_*α*_, the application of Rule 4 gives rise to $H_{1}, \dots , H_{\beta -1}$ with *E*(*H*_*i*_) = *T*_*i*_ ∪{*e*_2_} for every $i \in \{1, \dots , \beta -1\}$.

It remains to consider the remaining [*e*_2_]-component *T*_*β*_ of *H*, but now restricted to the hypergraph *G*_*α*_ = *S*_*α*_ ∪{*e*_1_}. It suffices to show that (*S*_*α*_ ∩ *T*_*β*_) ∪{*e*_1_} is [*e*_2_]-connected because, in this case, we would indeed get (*S*_*α*_ ∩ *T*_*β*_) ∪{*e*_1_} as the remaining [*e*_2_]-component when applying Rule 4 to *G*_*α*_, and *K* with *E*(*K*) = (*S*_*α*_ ∩ *T*_*β*_) ∪{*e*_1_,*e*_2_} would be the remaining hypergraph produced by this application of Rule 4. The proof follows the same line of argumentation as Case 2.1. More specifically, assume to the contrary that there are two edges *f*_1_,*f*_2_ in (*S*_*α*_ ∩ *T*_*β*_) ∪{*e*_1_}, such that *f*_1_,*f*_2_ are not [*e*_2_]-connected in *G*_*α*_. However, *f*_1_,*f*_2_ are in the same [*e*_2_]-component *T*_*β*_ of *H*. Hence, there exists a path between *f*_1_ and *f*_2_ using an edge from some [*e*_1_]-component different from *S*_*α*_. This can be exploited to derive a contradiction by constructing an [*e*_1_]-path between two different [*e*_1_]-components. For details, see Case 2.1.

## Parallelisation strategy

As described in more detail below, we use a divide and conquer method, based on the balanced separator approach. This method divides a hypergraph into smaller hypergraphs, called *subcomponents*. Our method proceeds to work on these subcomponents in parallel, with each round reducing the size of the hypergraphs (i.e., the number of edges in each subcomponent) to at most half their size. Thus after logarithmically many rounds, the method will have decomposed the entire hypergraph, if a decomposition of width *k* exists. For the computation we use the modern programming language Go [10], which has a model of concurrency based on [28].

In Go, a *goroutine* is a sequential process. Multiple goroutines may run concurrently. In the pseudocode provided, these are spawned using the keyword **go**, as can be seen in Algorithm 1, line 16. They communicate over *channels*. Using a channel *ch* is indicated by $\leftarrow ch$ for *receiving* from a channel, and by $ch \leftarrow$ for *sending* to *ch*.

### Overview

Algorithm 1 contains the full decomposition procedure, whereas Function FindBalSep details the parallel search for separators, as it is a key subtask for parallelisation. To emphasise the core ideas of our parallel algorithm, we present it as a decision procedure, which takes as input a hypergraph *H* and a parameter *k*, and returns as output either *Accept* if *ghw*(*H*) ≤ *k* or *Reject* otherwise. Please note, however, that our actual implementation also produces a GHD of width ≤ *k* in case of an accepting run.

For the GHD computation, we may assume w.l.o.g. that the input hypergraph has no isolated vertices (i.e., vertices that do not occur in any edge). Hence, we may identify *H* with its set of edges *E*(*H*) with the understanding that $V(H) = \bigcup E(H)$ holds. Likewise, we may consider a subhypergraph $H^{\prime }$ of *H* as a subset $H^{\prime } \subseteq H$ where, strictly speaking, $E(H^{\prime }) \subseteq E(H)$ holds.

Our parallel Balanced Separator algorithm begins with an initial call to the procedure Decomp, as seen in line 2 of Algorithm 1. The procedure Decomp takes two arguments, a subhypergraph $H^{\prime }$ of *H* for the current subcomponent considered, and a set *Sp* of “special edges”. These special edges indicate the balanced separators encountered so far, as can be seen in line 16, where the current separator *subSep* is added to the argument on the recursive call, combining all its vertices into a new special edge. The special edges are needed to ensure that the decompositions of subcomponents can be combined to an overall decomposition, and are thus considered as additional edges. The goal of procedure Decomp is to find a GHD ${{\mathscr{D}}}$ of $H^{\prime } \cup Sp$ in such a way that each special edge *s* ∈ *Sp* must be “covered” by a leaf node *n*_*s*_ of ${{\mathscr{D}}}$ with the properties *λ*(*n*_*s*_) = {*s*} and *χ*(*n*_*s*_) = *s* and *s* may not occur in the *λ*-label of any other node in the GHD, i.e., we may only use edges from *H* for these *λ*-labels. Thus the set *Sp* imposes additional conditions on the GHD. In the sequel, we shall refer to a pair ($H^{\prime }, Sp)$ consisting of a subhypergraph $H^{\prime }$ of *H* and a set of special edges *Sp* as an “extended subhypergraph” of *H*. Clearly, also *H* itself together with the empty set of special edges is an extended subhypergraph of itself and a GHD of *H* also satisfies the additional conditions of a GHD of the extended subhypergraph (*H*,*∅*). Hence, the central procedure Decomp in Algorithm 1, when initially called on line 2, checks if there exists a GHD of desired width of the extended subhypergraph (*H*,*∅*), that is, a GHD of hypergraph *H* itself.

The recursive procedure Decomp has its base case in lines 4 to 5, when the size of $H^{\prime }$ and *Sp* together is less than or equal to 2. The remainder of Decomp consists of two loops, the Separator Loop, from lines 7 to 24, which iterates over all balanced separators, and within it the SubEdge Loop, running from lines 12 to 23, which iterates over all subedge variants of any balanced separator. New balanced separators are produced with the subprocedure FindBalSep, used in line 8 of Algorithm 1, and detailed in Function FindBalSep. After a separator is found, Decomp computes the new subcomponents in line 13. Then goroutines are started using recursive calls of Decomp for all found subcomponents. If any of these calls returns Reject, seen in line 19, then the procedure starts checking for subedges. If they have been exhausted, the procedure checks for another separator. If all balanced separators have been tried without success, then Decomp rejects in line 25.

We proceed to detail the parallelisation strategy of the two key subtasks: the search for new separators and the recursive calls over the subcomponents created from a chosen separator.

### Parallel search for balanced separators

Before describing our implementation, we define some needed notions. For the search for balanced separators within an extended subhypergraph $H^{\prime } \cup Sp$, we can determine the set of relevant edges from the hypergraph, defined as $E^{*} = \{ e \in E(H) \mid e \cap \bigcup (H^{\prime } \cup Sp) \not = \emptyset \}$. We assume for this purpose that the edges in *E*^∗^ are ordered and carry indices in $\{1, \dots , |E^{*}|\}$ according to this ordering. We can then define the following notion.

#### Definition 1

A *k-combination* for an ordered set of edges *E*^∗^ is a *k*-tuple of integers $(x_{1}, \dots , x_{k})$, where 1 ≤ *x*_*i*_ ≤|*E*^∗^| and $x_{1} < {\dots } < x_{k}$. For two *k*-combinations *a*,*b*, we say *b* is one step ahead of *a*, denoted as *a* <_1_*b*, if w.r.t. the lexicographical ordering <_*lex*_ on the tuples, we have *a* <_*lex*_*b*, and there exists no other *k*-combination *c* s.t. *a* <_*lex*_*c* <_*lex*_*b*. To generalise, we say *c* is *i* steps ahead of *a* with *i* > 1, if there exists some *b* s.t. *a* <_*i*− 1_*b* <_1_*c*.

#### Example 3

**Fig. 3:**
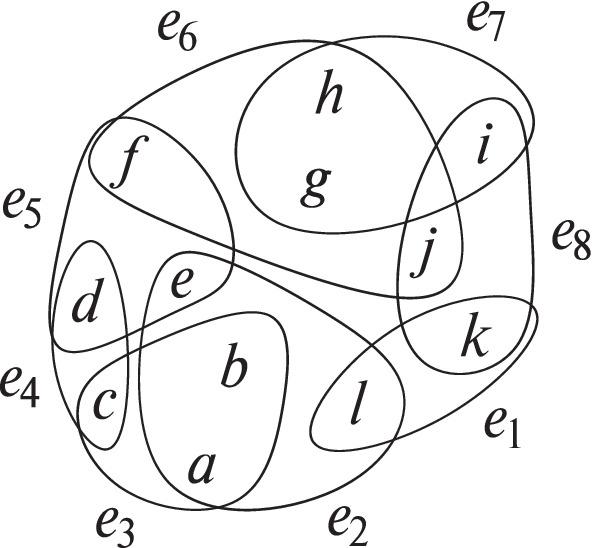
An example hypergraph, where the vertices are represented by letters, with explicit edge names

Consider the hypergraph *H* from Fig. 3. Assume that we are currently investigating the extended subhypergraph with $H^{\prime } = \{ e_{3},e_{4},e_{5}\}$ and *Sp* = {{*a*,*b*,*e*,*f*}}. By the definition above, this gives us the following set of relevant edges: *E*^∗^ = {*e*_2_,*e*_3_,*e*_4_,*e*_5_,*e*_6_}. We assume the ordering to be simply the order the edges are written in here, i.e., index 1 refers to edge *e*_2_, 2 refers to *e*_3_, etc.

Let us assume that we are looking for separators of length 3, so *k* = 3. We would then start the search with the 3-combination (1,2,3), which represents the choice of *e*_2_,*e*_3_,*e*_4_. If we move one step ahead, we next get the 3-combination (1,2,4), which represents the choice of *e*_2_,*e*_3_,*e*_5_. Moving further 3 steps ahead, we produce the 3-combination (1,3,5), representing the choice of *e*_1_,*e*_4_,*e*_6_.

In our parallel implementation, while testing out a number of configurations, we settled ultimately on the following scenario, shown in Function FindBalSep: we first create *w* many worker goroutines, seen in lines 3 to 4, where *w* stands for the number of CPUs we have available. This corresponds to splitting the workspace into *w* parts, and assigning each of them to one worker. Each worker is passed two arguments:

First, the workers are passed a channel *ch*, which they will use to send back any balanced separators they find. The worker procedure iterates over all candidate separators in its assigned search space, and sends back the first balanced separator it finds over the channel.

**Fig. 4:**
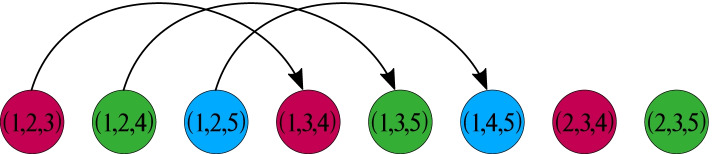
Using *k*-combinations to split the workspace. Shown here with 3 workers, and *k* = 3 and |*E*^∗^| = 5

Secondly, to coordinate the search, each worker is passed a *k-combination*, where the needed ordering is on the relevant edges defined earlier. Furthermore, each worker starts with a distinct offset of *j* steps ahead, where 0 ≤ *j* ≤ *w* − 1, and will only check *k*-combinations that are *w* steps apart each. This ensures that no worker will redo the work of another one, and that together they still cover the entire search space. An illustration for this can be seen in Fig. 4.

Having started the workers, FindBalSep then waits for one of two conditions (whichever happens first): either one of the workers finds a balanced separator, lines 6 to 7, or none of them does and they all terminate on their own once they have exhausted the search space. Then the empty set is returned, as seen in line 8, indicating that no further balanced separators from edges in *E*^∗^ exist. We note that balanced separators composed from subedges are taken care of in Algorithm 1 on lines 19 to 21, and are therefore not relevant for the search inside the Function FindBalSep.

We proceed to explain how this design addresses the three challenges for a parallel implementation we outlined in the introduction.

i This design reduces the need for synchronisation: each worker is responsible for a share of the search space, and the only time a worker is stopped is when either it has found a balanced separator, or when another worker has done so.
ii The number of worker goroutines scales with the number of available processors. This allows us to make use of the available hardware when searching for balanced separators, and the design above makes it very easy to support an arbitrary number of processors for this, without a big increase in the synchronisation overhead.
iii Finally, our design addresses backtracking in this way: as explained, the workers employ a set of *k*-combinations, called M in Function FindBalSep, to store their current progress, allowing them to generate the next separator to consider. Crucially, this data structure is stored in Decomp, seen in line 6 of Algorithm 1,*even after the search is over*. Therefore, in case we need to backtrack, this allows the algorithm to quickly continue the search exactly where it left off, without losing any work. If multiple workers find a balanced separator, one of them arbitrarily “wins”, and during backtracking, the other workers can immediately send their found separators to FindBalSep again.

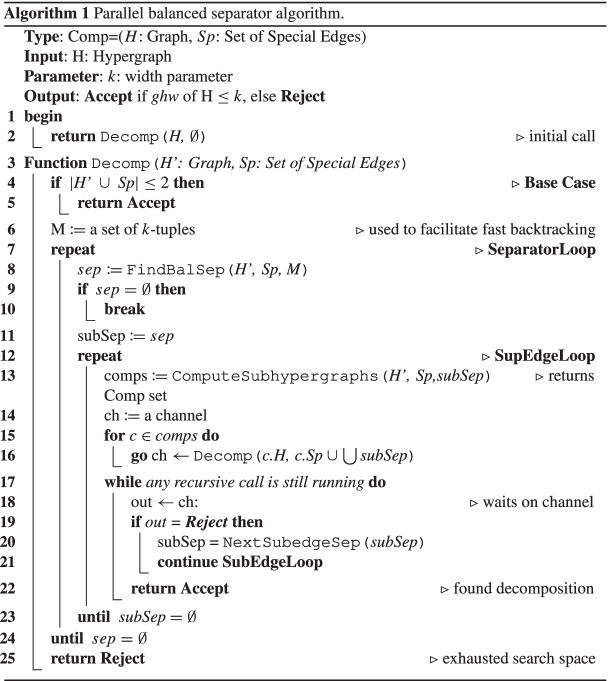

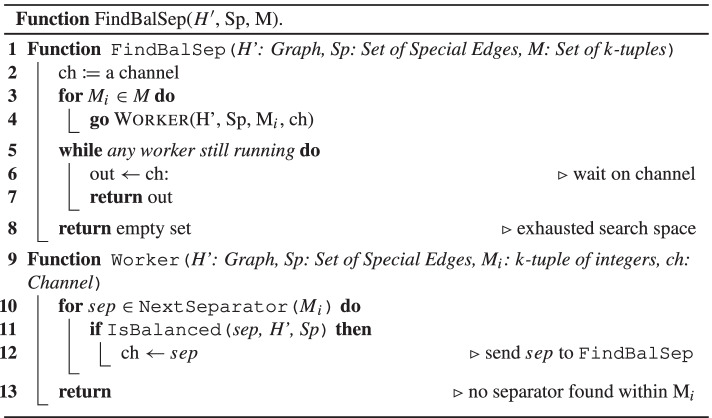


### Parallel recursive calls

For the recursive calls on the produced subcomponents, we create for each such call its own goroutine, as explained in the overview. This can be seen in Algorithm 1, line 15, where the output is then sent back via the channel *ch*. Each call gets as arguments its own extended subhypergraph, as well as an additional special edge. The output is received at line 18, where the algorithm waits on all recursive calls to finish before it can either return accept, or reject the current separator in case any recursive call returns a reject.

The fact that all recursive calls can be worked on concurrently is also in itself a major performance boost: in the sequential case we execute all recursive calls in a loop, but in the parallel algorithm - see lines 14 to 15 in Algorithm 1 - we can execute these calls simultaneously. Thus, if one parallel call rejects, we can stop all the other calls early, and thus potentially save a lot of time. It is easy to imagine a case where in the sequential execution, a rejecting call is encountered only near the end of the loop.

We state how we addressed the challenges of parallelisation in this area:

i Making use of goroutines and channels makes it easy to avoid any interference between the recursive calls, and the design allows each recursive call to run and return its results fully independently. Thus when running the recursive calls concurrently, we do not have to make use of synchronisation at all.
ii The second challenge, scaling with the number of CPUs, is initially limited by the number of recursive calls, which itself is dependent on the number of connected components. We can ensure, however, that we will generally have at least two, unless we manage to cover half the graph with just *k* edges. While this might look problematic at first, each of these recursive calls will either hit a base case, or once more start a search for a balanced separator which as outlined earlier, will always be able to make use of all cores in our CPU. This construction is aided by the fact that Go can easily manage a very large number of goroutines, scheduling them to make optimal use of the available resources. Thus our second challenge has also been addressed.
iii The third challenge, regarding backtracking, was written with the search for a balanced separator in mind, and is thus not directly applicable to the calls of the procedure Decomp. To speed up backtracking also in this case, we did initially consider the use of caching – which was used to great effect in det-*k*-decomp [23]. The algorithm presented here, however, differs significantly from det-*k*-decomp by the introduction of special edges. This makes cache hits very unlikely, since both the subhypergraph $H^{\prime }$ and the set of special edges *Sp* must coincide between two calls of Decomp, to reuse a previously computed result from the cache. Hence, caching turned out to be not effective here.

Another important topic concerns the scheduling of goroutines. This is relevant for us, since during every recursive call, we start as many goroutines as there are CPUs. Luckily, Go implements a so-called “work stealing” scheduler, which allows idle CPUs to take over parts of the work of other CPUs. Since goroutines have less of an overhead than normal threads, we can be sure that our algorithm maximally utilises the given CPU resources, without creating too much of an overhead. For more information about the scheduling of goroutines, we refer to the handbook by Cox-Buday [9].

To summarise, two of the challenges were addressed and solved, while the third, which mainly targeted the search for a balanced separator, was not applicable here. The parallelisation of recursive calls therefore gives a decent speed-up as will be illustrated by the experimental results in Section 5.

### Correctness of the parallel algorithm

It is important to note that this parallel algorithm is a correct decomposition procedure. More formally, we state the following property:

#### Theorem 3

The algorithm for checking the *ghw* of a hypergraph given in Algorithm 1 is sound and complete. More specifically, Algorithm 1 with input *H* and parameter *k* will accept if and only if there exists a GHD of *H* with width ≤ *k*. Moreover, by materialising the decompositions implicitly constructed in the recursive calls of the Decomp function, a GHD of width ≤ *k* can be constructed efficiently in case the algorithm returns Accept.

#### Proof

A sequential algorithm for GHD computation based on balanced separators was first presented in [14]. Let us refer to it as SequentialBalSep. A detailed proof of the soundness and completeness of SequentialBalSep is given in [14]. For convenience of the reader, we recall the pseudo-code description of SequentialBalSep from [14] in Appendix A. In order to prove the soundness and completeness of our parallel algorithm for GHD computation, it thus suffices to show that, for every hypergraph *H* and integer *k* ≥ 1, our algorithm returns Accept if and only if SequentialBalSep returns a GHD of *H* of width ≤ *k*. Hence, since both algorithms operate on the same notion of extended subhypergraphs and their GHDs, we have to show that, for every *k* ≥ 1 and every input $(H^{\prime },Sp)$, the Decomp function of our algorithm returns Accept if and only if the Decompose function of the SequentialBalSep algorithm returns a GHD of $H^{\prime } \cup Sp$ of width ≤ *k*.

To prove this equivalence between our new parallel algorithm and the previous SequentialBalSep algorithm from [14], we inspect the main differences between the two algorithm and argue that they do not affect the equivalence:

1. *Decision problem vs. search problem.* While SequentialBalSep outputs a concrete GHD of desired width if it exists, we have presented our algorithm as a pure decision procedure which outputs Accept or Reject. Note that this was only done to simplify the notation. It is easy to verify that the construction of a GHD in the SequentialBalSep algorithm on lines 5 – 12 (for the base case) and on line 27 (for the inductive case) can be taken over literally for our parallel algorithm.
2. *Parallelisation.* The most important difference between the previous sequential algorithm and the new parallel algorithm is the parallelisation. As was mentioned before, parallelisation is applied on two levels: splitting the search for finding the next balanced separator into parallel subtasks via function FindBalSep and processing recursive calls of function Decomp in parallel. The parallelisation via function FindBalSep will be discussed separately below. We concentrate on the recursive calls of function Decomp first. On lines 13 – 22 of our parallel algorithm, function Decomp is called recursively for all components of a given balanced separator and Accept is returned on line 22 if and only if all these recursive calls are successful. Otherwise, the next balanced separator is searched for. The analogous work is carried out on lines 18 – 27 of the SequentialBalSep algorithm. That is, the function Decompose is called recursively for all components of a given balanced separator and (by combining the GHDs returned from these recursive calls) a GHD of the given extended subhypergraph is returned on line 27 if and only if all these recursive calls are successful. Otherwise, the next balanced separator is searched for.
3. *Search for balanced separators.* As has been detailed in Section 4.2, our function FindBalSep splits the search space for a balanced separator into *w* pieces (where *w* denotes the number of available workers) and searches for a balanced separator in parallel. So in principle, this function has the same functionality as the iterator BalSepIt in the SequentialBalSep algorithm. That is, the set of balanced separators of size *k* for an extended subhypergraph $H^{\prime } \cup Sp$ found is the same when one calls the function FindBalSep until it returns the empty set, or when one calls the iterator BalSepIt until it has no elements to return any more. However, the calls of function FindBalSep implement one of the algorithmic improvements presented in Section 3.2: note that the SequentialBalSep algorithm assumes that all required subedges of edges from *E*(*H*) have been added to *E*(*H*) before executing this algorithm. It may thus happen that, by considering different subedges of a given *k*-tuple of edges, the same separator (i.e., the same set of vertices) is obtained several times. As has been explained in Section 3.2, we avoid this repetition of work by concentrating on the set of vertices of a given balanced separator (i.e., sep returned on line 8 and used to initialize subSep on line 11) and iterate through all balanced separators obtained as “legal” subsets by calling the NextSubedgeSep function on line 20. This means that we ultimately get precisely the same balanced separators (considered as sets of vertices) as in the SequentialBalSep algorithm.

### Hybrid approach - best of both worlds

Based on this parallelisation scheme, we produced a parallel implementation of the Balanced Separator algorithm, with the improvements mentioned in Section 3. We already saw some promising results, but we noticed that for many instances, this purely parallel approach was not fast enough. We thus continued to explore a more nuanced approach, mixing both parallel and sequential algorithms.

We now present a novel combination of parallel and sequential decomposition algorithms. It contains all the improvements mentioned in Section 3 and combines the Balanced Separator algorithm from Sections 4.1–4.3 and det-*k*-decomp recalled in Section 2.1.

This combination is motivated by two observations: The Balanced Separator algorithm is very effective at splitting large hypergraphs into smaller ones and in negative cases, where it can quickly stop the computation if no balanced separator for a given subcomponent exists. It is slower for smaller instances where the computational overhead to find balanced separators at every step slows things down. Furthermore, for technical reasons, it is also less effective at making use of caching. det-*k*-decomp, on the other hand, with proper heuristics, is very efficient for small instances and it allows for effective caching, thus avoiding repetition of work.

The Hybrid approach proceeds as follows: For a fixed number *m* of rounds, the algorithm tries to find balanced separators. Each such round is guaranteed to halve the number of hyperedges considered. Hence, after those *m* rounds, the number of hyperedges in the remaining subcomponents will be reduced to at most $\frac {|E(H)|}{2^{m}}$. The Hybrid algorithm then proceeds to finish the remaining subcomponents by using the det-*k*-decomp algorithm.

This required quite extensive changes to det-*k*-decomp, since it must be able to deal with Special Edges. Formally, each call of det-*k*-decomp runs sequentially. However, since the *m* rounds can produce a number of components, many calls of det-*k*-decomp can actually run in parallel. In other words, our Hybrid approach also brings a certain level of parallelism to det-*k*-decomp.

## Experimental evaluation and results

We have performed our experiments on the HyperBench benchmark from [14] with the goal to determine the exact generalized hypertree width of significantly more instances. We thus evaluated how our approach compares with existing attempts to compute the *ghw*, and we investigated how various heuristics and parameters prove beneficial for various instances. The detailed results of our experiments^1^, in addition to the source code of our Go programs^2^ are publicly available. Together with the benchmark instances, which are detailed below and also publicly available, this ensures the reproducibility of our experiments.

### Benchmark instances and setting

#### HyperBench

The instances used in our experiments are taken from the benchmark HyperBench, collected from various sources in industry and the literature, which was released in [14] and made publicly available at http://hyperbench.dbai.tuwien.ac.at. It consists of 3648 hypergraphs from CQs and CSPs, where for many CSP instances the exact *ghw* was still undetermined. In this extended evaluation, we performed the evaluation on a larger set of instances when compared with the original paper [21], to reflect the newest version of the benchmark, published in [14]. We provide a more detailed overview of the various instances, grouped by their origin, in Table 2. The first two columns of “Avg sizes”, refer to the sizes of instances within the groups, and the final column “Size” refers to the cardinality of the group, i.e. how many instances it includes. The two “Arity” columns refer to the maximum and average edge sizes of the hypergraphs in each group.

**Table 2:** Overview of the instances from HyberBench and their average sizes by group, as well as sizes of groups themselves

Instances					
Group	Avg sizes		Arity		Size
	|*V* |	|*E*|	avg	max	
CSP Application	151.71	68.90	7.00	35	1090
CSP Random	40.74	67.58	4.85	15	863
CSP Other	372.40	395.68	4.24	14	82
CQ Application	30.88	7.03	11.03	145	1113
CQ Random	47.99	27.54	10.63	20	500
Total	79.34	51.39	8.15	145	3648

#### Hardware and software

We used Go 1.2 for our implementation, which we refer to as *BalancedGo*. Our experiments ran on a cluster of 12 nodes, running Ubuntu 16.04.1 LTS with a 24 core Intel Xeon E5-2650v4 CPU, clocked at 2.20 GHz, each node with 256 GB of RAM. We disabled HyperThreading for the experiments.

#### Setup and limits

For the experiments, we set a number of limits to test the efficiency of our solution. For each run, consisting of the input (i.e., hypergraph *H* and integer *k*) and a configuration of the decomposer, we set a one hour (3600 seconds) timeout and limited the available RAM to 1 GB. These limits are justified by the fact that these are the same limits as were used in [14], thus ensuring the direct comparability of our test results. To enforce these limits and run the experiments, we used the *HTCondor* software [39], originally named just Condor. Note that for the test results of HtdSMT, we set the available RAM to 24 GB, as that particular solver had a much higher memory consumption during our tests.

### Empirical results

The key results from our experiments are summarised in Table 4, with Table 3 acting as a comparison point. Under “Decomposition Methods” we use “*ensemble*” to indicate that results from multiple algorithms are collected, i.e., results from the Hybrid algorithm, the parallel Balanced Separator algorithm and det-*k*-decomp. To also consider the performance of one of the individual approaches introduced in Section 4, namely the results of the Hybrid approach (from Section 4.5) is separately shown in a section of the table. As a reference point, we considered on one hand the *NewDetKDecomp* library from [14] and also the SAT Modulo Theory based solver *HtdSMT* from [38]. For each of these, we also listed the average time and the maximal time to compute a GHD of optimal-width for each group of instances of HyperBench, as well as the standard deviation. The minimal times are left out for brevity, since they are always near or equal to 0. Note that for HyberBench the instance groups “CSP Application” or “CQ Application”, listed in Tables 3 and 4 are hypergraphs of (resp.) CSP or CQ instances from real world applications.

**Table 3:** Overview of previous results: number of instances solved and running times (in seconds) for producing optimal-width GHDs in [14] and [38]

Instances	Decomposition Methods							
Group	NewDetKDecomp by [14]				HtdSMT by [38]			
	#solved	avg	max	stdev	#solved	avg	max	stdev
CSP Application	386	150.82	2608.0	490.47	571	227.27	3508.5	529.90
CSP Random	412	65.78	3240.0	379.12	587	366.93	3569.0	756.10
CSP Other	27	126.43	2538.0	422.42	23	371.77	3340.3	728.27
CQ Application	1113	0.00	0.0	0.00	1070	32.00	1437.0	113.60
CQ Random	281	2.12	335.0	21.14	254	192.30	3486.5	552.20
Total	2219	59.00	3240.0	325.03	2505	158.25	3569.0	481.64

**Table 4:** Overview of our results: number of instances solved and running times (in seconds) for producing optimal-width GHDs by our new algorithms

Instances	Decomposition Methods							
Group	Hybrid Approach				BalancedGo *ensemble*			
	#solved	avg	max	stdev	#solved	avg	max	stdev
CSP Application	762	6.24	3247.90	80.70	763	30.86	3572.78	211.83
CSP Random	578	29.31	3589.82	246.19	625	48.60	3589.82	297.21
CSP Other	42	34.33	2236.00	194.52	42	45.86	2438.64	223.75
CQ Application	1113	0.00	0.01	0.00	1113	0.00	1.74	0.02
CQ Random	355	16.45	3574.76	198.97	381	27.87	3574.76	231.01
Total	2850	11.30	3589.82	145.47	2924	25.76	3589.82	207.32

In the left part of Table 4, we report on the following results obtained with our Hybrid Approach described in Section 4.5, while the right part of that table shows the result for the “BalancedGo ensemble”. Recall that by “ensemble” we mean the combination of the information gained from runs of all our decomposition algorithms. For a hypergraph *H* and a width *k*, an accepting run gives us an upper bound (since the optimal *ghw*(*H*) is then clearly ≤ *k*), and a rejecting run gives us a lower bound (since then we know that *ghw*(*H*) > *k*). By pooling multiple algorithms, we can combine these produced upper and lower bounds to compute the optimal width (when both bounds meet) for more instances than any one algorithm could determine on its own. We note that the results for NewDetKDecomp from Fischl et al. [14] are also such an “ensemble”, combining the results of three different GHD algorithms presented in [14]. Across all experiments, out of the 3648 instances in HyperBench, we have thus managed to solve over 2900. By “solved” we mean that the precise *ghw* could be determined in these cases. It is interesting to note that the Hybrid Algorithm on its own is almost as good as the “ensemble”. Indeed, the number of 2924 solved instances in case of the “ensemble” only mildly exceeds the the number of 2850 instances solved by the implementation of our Hybrid algorithm. The strength of the Hybrid algorithm stems from the fact that it combines the ability of the parallel Balanced Separator approach for quickly deriving lower bounds (i.e., detecting “Reject”-cases) with the ability of det-*K*-decomp for more quickly deriving upper bounds (i.e., detecting “Accept”-cases).

**Fig. 5:**
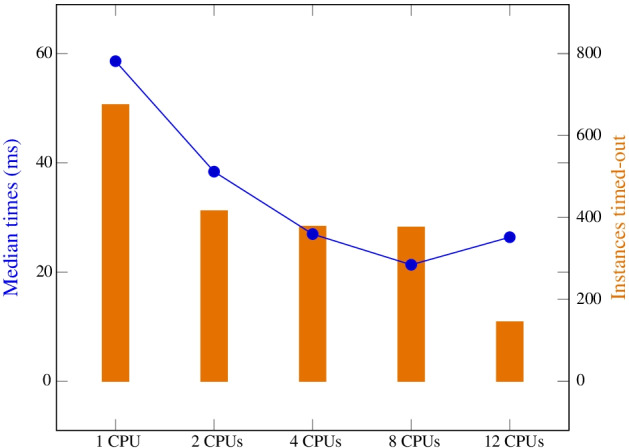
Study of the performance gain w.r.t. the number of CPUs used

Figure 5 shows runtimes for all positive runs of the Hybrid algorithm over all instances of HyperBench with an increasing number of CPUs used, where the used width parameter ranges from 2 to 5. The blue dots signify the median times in milliseconds, and the orange bars show the number of instances which produced timeouts. We can see that increasing the CPUs either reduces the median (solving the same instances faster) or reduces the number of instances which timed out. Actually, reducing the number of time-outs is potentially a much higher speedup than merely reducing the median, and also of higher practical interest, as it allows us to decompose more instances in realistic time. It should be noted that the increase of the median time when we go from 8 CPUs to 12 CPUs does not mean at all that the performance degrades due to the additional CPUs. The additional time consumption is solely due to the increased number of solved instances, which are typically hard ones. And the computation time needed to solve them enters the statistics only if the computation does not time out (Table 5).

**Table 5:** Comparison of BalancedGo, HtdSMT and TULongo on the PACE 2019 Challenge, Track 2a

Method	# of solved instances	# of solved private instances	*t*_avg_ (sec)	*t*_sum_ (h)
BalancedGo	172	86	134.24	3.21
HtdSMT	165	80	128.67	2.89
TULongo [34]	70	38	105.58	1.11

Columns *t*_avg_ and *t*_sum_ show the average time and the total time, respectively, over all private instances

In order to fully compare the strengths and weaknesses of each of the discussed decomposition methods, we also investigated the number of instances that could only be solved via a specific approach. This can be seen in Table 6. We see that while our approach clearly dominates this metric, there are still many cases where other methods were more effective.

**Table 6:** Overview of exclusively solved instances of HyberBench for each decomposition method

Method	#exclusively solved
BalancedGo *ensemble*	284
NewDetKDecomp	11
HtdSMT	67

**Fig. 6:**
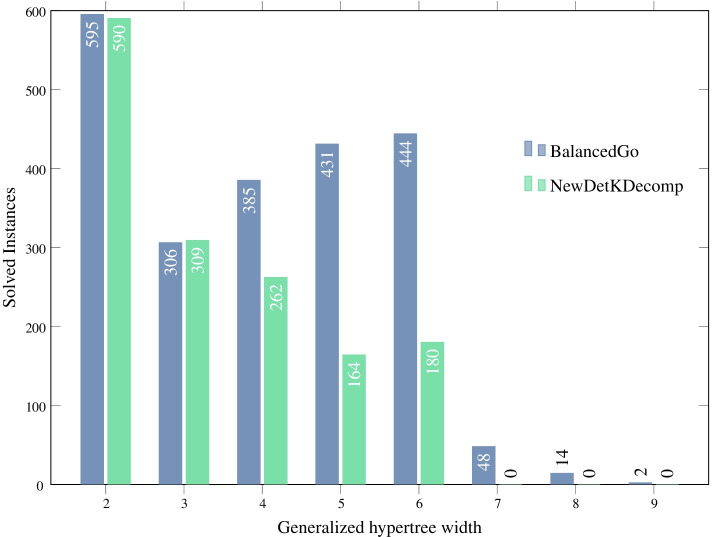
Overview of the distribution of the *ghw* of the solved instances

In Fig. 6 we see an overview of the distribution of the *ghw* of all solved instances of our approach, and as a comparison we see how many instances for each width could be determined by NewDetKDecomp.

For the computationally most challenging instances of HyperBench, those of *ghw*≥ 3, our result signifies an increase of over 70 % in solved instances when compared with [14]. In addition, when considering the CSP instances from real world applications, we managed to solve 763 instances, almost doubling the number from NewDetKDecomp. In total, we now know the exact *ghw* of around 70% of all hypergraphs from CSP instances and the exact *ghw* of around 75% of all hypergraphs of HyperBench.

Another aspect of our solver we wanted to explore was the memory usage, and whether lifting the restriction to merely 1 GB of RAM makes a difference in the number of GHDs that can be found. We therefore looked at all test runs of lower width, ≤ 5 where our solver timed out. There were 91 such instances. This restriction seems justified as the width parameter affects the complexity of determining the *ghw* exponentially, thus it is only for lower widths that one would expect memory to become a limiting factor as opposed to time. We reran these 91 test instances using 24 GB of RAM. It turned out that the increase in available memory made no difference, however, as all 91 tests still timed out. In other words, the limiting factor in computing hypergraph decompositions is time, not space.

We stress that, in the first place, our data in Table 4 is not about time, but rather about the number of instances solved within the given time limit of 1 hour. And here we provide an improvement for these practical CSP instances of near 100% on the current state of the art; no such improvements have been achieved by other techniques recently. It is also noteworthy, that the Hybrid algorithm alone solved 2850 total cases, thus beating the total for NewDetKDecomp in [14], which, as mentioned, combines the results of three different GHD algorithms and also beating the total for HtdSMT [38].

#### Comparison with PACE 2019 Challenge

In addition to experiments on HyperBench, we also compared our implementation with various solvers presented during the PACE 2019 Challenge [11], where one track consisted in solving the exact hypertree width. We took the 100 public and 100 private instances from the challenge (themselves a subset of HyperBench), and tried to compute the exact *ghw* of the instances within 1800 seconds, using at most 8 GB of RAM. Since our test machine is different from the one used during PACE 2019 Challenge, we took the implementations of the winner and runner up, HtdSMT and TULongo [34] and reran them again using the same time and memory constraints. The results can be seen in Table 5. *BalancedGo* managed to compute 86 out of the 100 private instances, improving slightly on HtdSMT. It is noteworthy that this was accomplished while computing GHDs, instead of the simpler HDs which were asked for during the challenge.

## Conclusion and outlook

We have presented several generally applicable algorithmic improvements for hypergraph decomposition algorithms and a novel parallel approach to computing GHDs. We have thus advanced the ability to compute GHDs of a significantly larger set of CSPs than previous GHD algorithms. This paves the way for more applications to use them to speed up the evaluation of CSP instances.

For future work, we envisage several lines of research: first, we want to further speed up the search for a first balanced separator as well as the search for a next balanced separator in case the first one does not lead to a successful decomposition. Note that for computing any *λ*-label of a node in a GHD of width ≤ *k*, in principle, *O*(*n*^*k*+ 1^) combinations of edges have to be investigated for |*E*(*H*)| = *n*. However, only a small fraction of these combinations is actually a balanced separator, leaving a lot of potential for speeding up the search for balanced separators. Apart from this important practical aspect, it would also be an interesting theoretical challenge to prove some useful upper bound on the ratio of balanced separators compared with the total number of possible combinations of up to *k* edges.

So far we were focused on the efficient and parallel computation of GHDs via balanced separators. It would be interesting to explore a similar approach for the computation of hypertree decompositions (HDs) [19]. The big advantage of HDs over GHDs is that their computation is tractable (for fixed *k*) even without adding certain subedges. On the other hand, HDs require the root of the decomposition to be fixed. In contrast, a GHD can be rooted at any node and the GHD algorithm via balanced separators crucially depends on the possibility of re-rooting subtrees of a decomposition. Significantly new ideas are required to avoid such re-rooting in case of HDs. First preliminary steps in this direction have already been made in [3] but many further steps are required yet.

In this work, we have looked at the decomposition of (the hypergraphs underlying) CSPs. The natural next step is to apply decompositions to actually solving CSP. Hence, another interesting goal for future research is harnessing Go’s excellent cloud computing capabilities to extend our results beyond the computation of GHDs to actually evaluating large real-life CSPs in the cloud.

## Data Availability

All the data used in our experimental evaluation has been published in an open access format (see [22]).

## Footnotes

1 raw data available under [22]

2 See: https://github.com/cem-okulmus/BalancedGo

## Code availability

The source code of our implementation is publicly available under https://github.com/cem-okulmus/BalancedGo.
